# Identified eleven exon variants in *PKD1* and *PKD2* genes that altered RNA splicing by minigene assay

**DOI:** 10.1186/s12864-023-09444-9

**Published:** 2023-07-19

**Authors:** Xuyan Liu, Xiaomeng Shi, Qing Xin, Zhiying Liu, Fengjiao Pan, Dan Qiao, Mengke Chen, Yiyin Zhang, Wencong Guo, Changying Li, Yan Zhang, Leping Shao, Ruixiao Zhang

**Affiliations:** 1 Department of Nephrology, the Affiliated Qingdao Municipal Hospital of Qingdao University, No.5 Donghai Middle Road, Qingdao, 266071 China; 2 Renal Division, Peking University First Hospital, Beijing, China; 3 Department of Nephrology, Dalian Medical University, Dalian, China; 4 Department of Nephrology, Shandong First Medical University, Taian, China; 5 Department of Nephrology, Weifang Medical University, Weifang, China; 6 Department of Emergency, the Affiliated Qingdao Municipal Hospital of Qingdao University, No.5 Donghai Middle Road, Qingdao, 266071 China

**Keywords:** *PKD1*, *PKD2*, Minigene assay, pre-mRNA splicing, Exonic variant, Exon skipping

## Abstract

**Background:**

Autosomal dominant polycystic kidney disease (ADPKD) is a common monogenic multisystem disease caused primarily by mutations in the *PKD1* gene or *PKD2* gene. There is increasing evidence that some of these variants, which are described as missense, synonymous or nonsense mutations in the literature or databases, may be deleterious by affecting the pre-mRNA splicing process.

**Results:**

This study aimed to determine the effect of these *PKD1* and *PKD2* variants on exon splicing combined with predictive bioinformatics tools and minigene assay. As a result, among the 19 candidate single nucleotide alterations, 11 variants distributed in *PKD1* (c.7866C > A, c.7960A > G, c.7979A > T, c.7987C > T, c.11248C > G, c.11251C > T, c.11257C > G, c.11257C > T, c.11346C > T, and c.11393C > G) and *PKD2* (c.1480G > T) were identified to result in exon skipping.

**Conclusions:**

We confirmed that 11 variants in the gene of *PKD1* and *PKD2* affect normal splicing by interfering the recognition of classical splicing sites or by disrupting exon splicing enhancers and generating exon splicing silencers. This is the most comprehensive study to date on pre-mRNA splicing of exonic variants in ADPKD-associated disease-causing genes in consideration of the increasing number of identified variants in *PKD1* and *PKD2* gene in recent years. These results emphasize the significance of assessing the effect of exon single nucleotide variants in ADPKD at the mRNA level.

**Supplementary Information:**

The online version contains supplementary material available at 10.1186/s12864-023-09444-9.

## Introduction

**Fig. 1:**
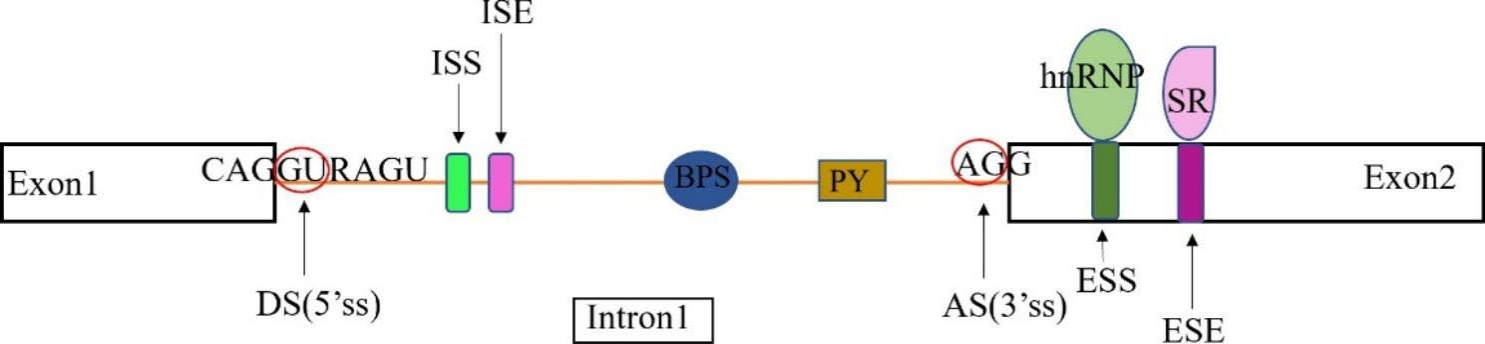
**The splicing sites in pre-mRNAs.** Abbreviations: donor site (DS), acceptor site (AS), branch point sequence (BPS), polypyrimidine tract (PY), exonic splicing enhancer (ESE), intronic splicing enhancer (ISE), exonic splicing silencer (ESS), intronic splicing silencer (ISS), serine/arginine-rich protein family (SR), heterologous nuclear ribonucleoprotein (hnRNP).

RNA splicing is a critical process in the posttranscriptional regulation of eukaryotic gene expression, where a newly-made precursor messenger RNA (pre-mRNA) transcript is transformed into a mature messenger RNA (mRNA). The removal of introns from the pre-mRNA is accomplished by the spliceosome, a large and highly dynamic ribonucleoprotein complex composed of five small nuclear ribonucleoprotein particles (snRNPs: U1, U2, U4, U5, and U6) [1]. It is estimated that about 50% of disease-associated single nucleotide variants (SNVs) affect the splicing pattern [2, 3]. The splicing process can be regulated by splicing signals to generate alternatively spliced mRNAs that encode diverse protein variants. Within introns, the donor site (DS, GU sequence at the 5′ end of the intron), the acceptor site (AS, AG sequence at the 3′ end of the intron), the branch point sequence (BPS, approximately 100 bp upstream of the 3′ end of the intron), and the polypyrimidine tract (PY, between the BPS and the 3′ end of the intron) are required for this process [4] (Fig. 1). The splicing signals also include cis-acting splicing regulatory elements, for example exonic/intronic splicing enhancers (ESEs/ISEs) and exonic/intronic splicing silencers (ESSs/ISSs) [5]. In addition, trans-acting splicing regulatory proteins, such as serine/arginine-rich protein family (SR) and heterologous nuclear ribonucleoproteins (hnRNPs), can promote or prevent the binding of snRNPs to the splice site by interacting with ESE/ISE or ESS/ISS, thus affecting splice site selection.(Fig. 1) Furthermore, RNA secondary structure is an important element in splicing regulation and its abnormalities can inhibit spliceosome assembly or interfere the binding of cis-acting elements and trans-acting factors [6].

Autosomal dominant polycystic kidney disease (ADPKD) is a common, monogenic, multi-system disease characterized by the development of multiple cysts in both kidneys and progressive chronic kidney disease with an estimated prevalence of between 1 and 1000 and 1 in 2500 individuals [7–9]. ADPKD is a systemic condition that can also be manifested as various extra-renal manifestations including hepatic and pancreatic cysts, abdominal hernias, heart valve disease, and intracranial aneurysms. Variants in the *PKD1* gene (MIM#601,313) and *PKD2* gene (MIM#173,910) are responsible for the most frequent cause of ADPKD, accounting for about 78% and 15% of cases, respectively [10]. Additionally, the alterations of some genes, such as *GANAB* (MIM#104,160) and *DNAJB11* (MIM#611,341), have an impact on the folding, maturation, and transport of *PKD1* and *PKD2* protein products, which may also contribute to the development of cysts [11–13].

*PKD1* gene is located on chromosome 16p13.3 with 46 exons that encode a 14 kb mRNA and *PKD2* is situated on chromosome 4q22.1 and contains 15 exons that generate a 3 kb mRNA [14]. Polycystic protein-1 (PC1) and polycystic protein-2 (PC2), transmembrane proteins encoded by *PKD1* and *PKD2*, form a functional complex that senses external chemical or mechanical stimuli, and regulate intracellular Ca^2+^ concentration [14].

DNA sequence analysis has been broadly recognized as an effective method to diagnose hereditary diseases. Over the past years the application of high-throughput sequencing techniques based on large-scale parallel sequencing has rapidly increased the number of identified variants. According to ADPKD Mutation Database (accessed February 2021, https://pkdb.mayo.edu/), 2322 *PKD1* and 278 *PKD2* variants have been reported, of which SNVs account for about 60% and 49%, respectively. However, single nucleotide substitutions accounted for approximately 51% and 44% of the 2499 *PKD1* and 399 *PKD2* different alterations described in the Human Gene Mutation Database [15] (HGMD, professional, accessed February 2021, http://www.hgmd.cf.ac.uk/ac/validate.php/), respectively. Approximately 55% of the variants in *PKD1* and *PKD2* are considered as be “pathogenic” or “likely pathogenic”, while many others are “uncertain significance” or “likely benign”. However, most variant analyses were designed to assess the effects on protein at the genome level, rather than the pathogenic changes caused by pre-RNA splicing, RNA folding, etc.

Fewer studies have identified splicing defects caused by exon variants as a pathogenesis of ADPKD [16]. The number of identified variants in *PKD1* and *PKD2* has increased significantly in recent years, especially the total number of alterations in *PKD1* rose from 416 to 2014 [17] to 1134 in 2021 (HGMD, professional, accessed February 2021). At present, the pathogenicity of the newly identified variants in *PKD1* and *PKD2* has not been comprehensive analyzed and verified in relevant literature. Therefore, herein we investigated the effect of SNVs in *PKD1* and *PKD2* on pre-mRNA splicing combined with bioinformatics tools and minigene assay.

## Result

Single nucleotide substitutions on the first and last exons were eliminated because they could not be analyzed with the minigene system. Francisco J has demonstrated that 3 variants of *PKD1* (327A > T, c.11156G > A and c.11257C > A) and 3 substitutions of *PKD2* (c.1532A > T, c.1716G > A and c.2657A > G) can result in incomplete or complete exon skipping by minigene analysis [18, 19]. Besides, the missense variant c.1320G > T [20] and nonsense variant c.2614C > T [21] have also been reported to alter pre-mRNA splicing, so these 8 single nucleotide substitutions were also excluded. Regulatory elements are known to be common in exons at weak splicing sites [22]. Therefore, we sought to select variants located in exons that have a weak 5′ or 3′ splice site predicted by Berkeley Drosophila Genome Project (BDGP). According to the screening criteria described in materials and methods, the following 19 candidate variants were enrolled in the study: 15 in *PKD1* (c.1202C > T, c.1248C > T, c.7866C > T c.7866C > A, c.7960A > G, c.7979A > T, c.7987C > T, c.11025G > A, c.11119C > T, c.11248C > G, c.11251C > T, c.11257C > G, c.11257C > T, c.11346C > T and c.11393C > G) and 4 in *PKD2* (c.741C > G, c.796G > T, c.1480G > T and c.1546G > T), as shown in Table 1. In addition, protein-level predictive analysis was performed of seven missense variants among these alterations (Table 2).

**Table 1:** Variants selected from this study in PKD1 and PKD2, and the results of silico analyses

Gene	Variants	Amino Acid	Exon (length)	Location in Exon	BDGP	ESEs Broken	New ESSs	△MaxEnt Donor site	△MaxEnt Acceptor site	rs ID
PKD1	c.1202C > T	p. Ala401Val	6(184)	+ 1	3′AS: 0.43→0.52	0	0		-27.22%	rs139917246
PKD1	c.1248C > T	p. Asn416=	6(184)	+ 47	NA	7	3		-2.71%	rs1303361524
PKD1	c.7866C > A	p. Tyr2622*	21(153)	+ 3	3′AS: 0.67→0.55	5	2		13.05%	rs181130940
PKD1	c.7866C > T	p. Tyr2622=	21(153)	+ 3	3′AS: 0.67→0.52	3	0			rs181130940
PKD1	c.7960A > G	p. Arg2654Gly	21(153)	-57	NA	5	9			NA
PKD1	c.7979A > T	p. Asp2660Val	21(153)	-38	NA	6	2		498.63%	NA
PKD1	c.7987C > T	p. Gln2663*	21(153)	-30	NA	8	2			rs1567182193
PKD1	c.11025G > A	p. Leu3675=	38(140)	+ 9	3′AS: 0.34→0.32	7	4			rs1430303850
PKD1	c.11119C > T	p. Gln3707*	38(140)	-38	NA	8	2	235.56%		rs2091668243
PKD1	c.11248C > G	p. Arg3750Gly	39(113)	-22	NA	9	5		36.36%	NA
PKD1	c.11251C > T	p. Gln3751*	39(113)	-18	NA	5	4	620%		NA
PKD1	c.11257C > G	p. Arg3753Gly	39(113)	-13	NA	3	10			rs1167476946
PKD1	c.11257C > T	p. Arg3753Trp	39(113)	-13	NA	1	8			rs1167476946
PKD1	c.11346 C > T	p. Asp3782=	40(142)	-64	NA	5	3			rs145955373
PKD1	c.11393C > G	p. Ser3798*	40(142)	-19	NA	3	7			NA
PKD2	c.741C > G	p. Tyr247*	3(134)	+ 36	NA	5	6			rs1578129049
PKD2	c.796G > T	p. Glu266*	3(134)	-48	NA	12	2		146.95%	NA
PKD2	c.1480G > T	p. Glu494*	6(229)	-69	NA	9	1			rs1727790591
PKD2	c.1546G > T	p. Val516Leu	6(229)	-3	5′DS: 0.62→0.68	2	6	-2.77%		rs143581690

Note: “=” indicates no amino acid changes after the gene variant (synonymous variant) and “*” represents a variant that becomes a termination code (nonsense variant);

“+” indicates distance from the 5′ end of the exon and “−” represents distance from the 3′ end.

AS, acceptor splice sites; DS, donor splice sites. ESE, exonic splicing enhancer; ESS, exonic splicing silencer; NA, not applicable.

Value 0, 1, 2, 3, 4, 5, and 6 indicate number of the splicing regulatory elements gained or disrupted.

ΔMaxEnt = MaxEnt Var − MaxEnt WT < 0 are assumed potential loss of 5′ donor or 3′ acceptor splice-site.

**Table 2:** Prediction of the potential pathogenicity of the missense variants

Gene	Variants	Amino Acid	Mutation Taster (score)	PolyPhen-2 (score)	ClinPred
PKD1	c.1202C > T	p. Ala401Val	polymorphism(0.926)	benign (0.000)	benign (0.001)
PKD1	c.7960A > G	p. Arg2654Gly	polymorphism(0.995)	benign (0.374)	probably damaging (0.516)
PKD1	c.7979A > T	p. Asp2660Val	disease causing(0.999)	probably damaging (1.000)	probably damaging (0.998)
PKD1	c.11248C > G	p. Arg3750Gly	disease causing(0.999)	probably damaging (0.981)	probably damaging (0.989)
PKD1	c.11257C > G	p. Arg3753Gly	disease causing(0.999)	benign (0.141)	probably damaging (0.997)
PKD1	c.11257C > T	p. Arg3753Trp	disease causing(0.999)	probably damaging (0.994)	probably damaging (0.998)
PKD2	c.1546G > T	p. Val516Leu	disease causing(0.966)	benign (0.000)	benign (0.014)

Seven control minigenes were separately constructed including *PKD1* wild-type (WT) sequences of exons 6 (pSPL3-*PKD1* Ex6), 20-21 (pSPL3-*PKD1* Ex20-21), 37-38 (pSPL3-*PKD1* Ex37-38), 39-40 (pSPL3-*PKD1* Ex39-40) and 40 (pSPL3-*PKD1* Ex40), and *PKD2* WT sequences of exons 3 (pSPL3-*PKD2* Ex3) and 6 (pSPL3-*PKD2* Ex6). All candidate variants minigenes were generated by site-directed mutagenesis based on the corresponding pSPL3-WT minigene. Unfortunately, these variants c.1202C > T, c.1248C > T, c.11025G > A, and c.11119C > T distributed in *PKD1* were not verifiable for technical reasons. Furthermore, we conducted a predictive analysis of the functional consequences of exon skipping caused by SNVs, and details are listed in Table 3.

**Table 3:** Prediction of the functional consequences of exon skipping caused by point mutations in this study

Gene	Variants	Type	Sites of Action	Splicing Changes	Frameshift	Protein
PKD1	c.7866C > A	nonsense	Splicing sites	partial skipping of exon21	in-frame deletions (codon 2622–2672)	51 aa loss in extracellular N-terminal domain (PC1)
PKD1	c.7960A > G	missense	ESEs/ESSs	partial skipping of exon21	in-frame deletions (codon 2622–2672)	51 aa loss in extracellular N-terminal domain (PC1)
PKD1	c.7979A > T	missense	ESEs/ESSs	partial skipping of exon21	in-frame deletions (codon 2622–2672)	51 aa loss in extracellular N-terminal domain (PC1)
PKD1	c.7987C > T	nonsense	ESEs/ESSs	partial skipping of exon21	in-frame deletions (codon 2622–2672)	51 aa loss in extracellular N-terminal domain (PC1)
PKD1	c.11248C > G	missense	ESEs/ESSs	partial skipping of exon39/ exon39 and exon40	p. Ser3720Thr fs*58/ in-frame deletions (codon 3720–3804)	truncated protein/ 84 aa loss in TOP domain (PC1)
PKD1	c.11251C > T	nonsense	ESEs/ESSs	partial skipping of exon39/ exon39 and exon40	p. Ser3720Thr fs*58/ in-frame deletions (codon 3720–3804)	truncated protein/ 84 aa loss in TOP domain (PC1)
PKD1	c.11257C > G	missense	ESEs/ESSs	partial skipping of exon39/ exon39 and exon40	p. Ser3720Thr fs*58/ in-frame deletions (codon 3720–3804)	truncated protein/ 84 aa loss in TOP domain (PC1)
PKD1	c.11257C > T	missense	ESEs/ESSs	partial skipping of exon39/ exon39 and exon40	p. Ser3720Thr fs*58/ in-frame deletions (codon 3720–3804)	truncated protein/ 84 aa loss in TOP domain (PC1)
PKD1	c.11346C > T	synonymous	ESEs/ESSs	partial skipping of exon40	p. Ala3757Gly fs*22	truncated protein (PC1)
PKD1	c.11393C > G	nonsense	ESEs/ESSs	partial skipping of exon40	p. Ala3757Gly fs*22	truncated protein (PC1)
PKD2	c.1480G > T	nonsense	Splicing sites and ESEs/ESSs	new alternative splicing and partial skipping of exon6	p. Cys476Ser fs*9 and p. Arg440Ser fs*5	truncated protein(PC2)

### Splicing outcome of sequences variations of *PKD1*

#### Exon 21

##### The variants that can alter splicing of exon 21

The nonsense variant (*PKD1*): c.7866C > A (p. Tyr2622X) is caused by a third nucleotide substitution on exon 21 of *PKD1*. Bioinformatic analysis demonstrated that this alteration reduced the score of acceptor site from 0.67 to 0.52 by BDGP and -32.48 to -33.36 by MaxEntScan [23, 24], respectively. Besides, the variant c.7866C > A was forecasted not only to disrupt five ESEs, but also to generate two ESSs by HSF 3.1 analysis. These missense variants (*PKD1*): c.7960A > G (p. Arg2654Gly), (*PKD1*): c.7979A > T (p. Asp2660Val) and nonsense variant (*PKD1*): c.7987C > T (p. Gln2663X) were located at the middle of exon 21 sequence, all of which resulted in the disruption of ESEs and the generation of ESSs according to HSF3.1 (Table 1).

**Fig. 2:**
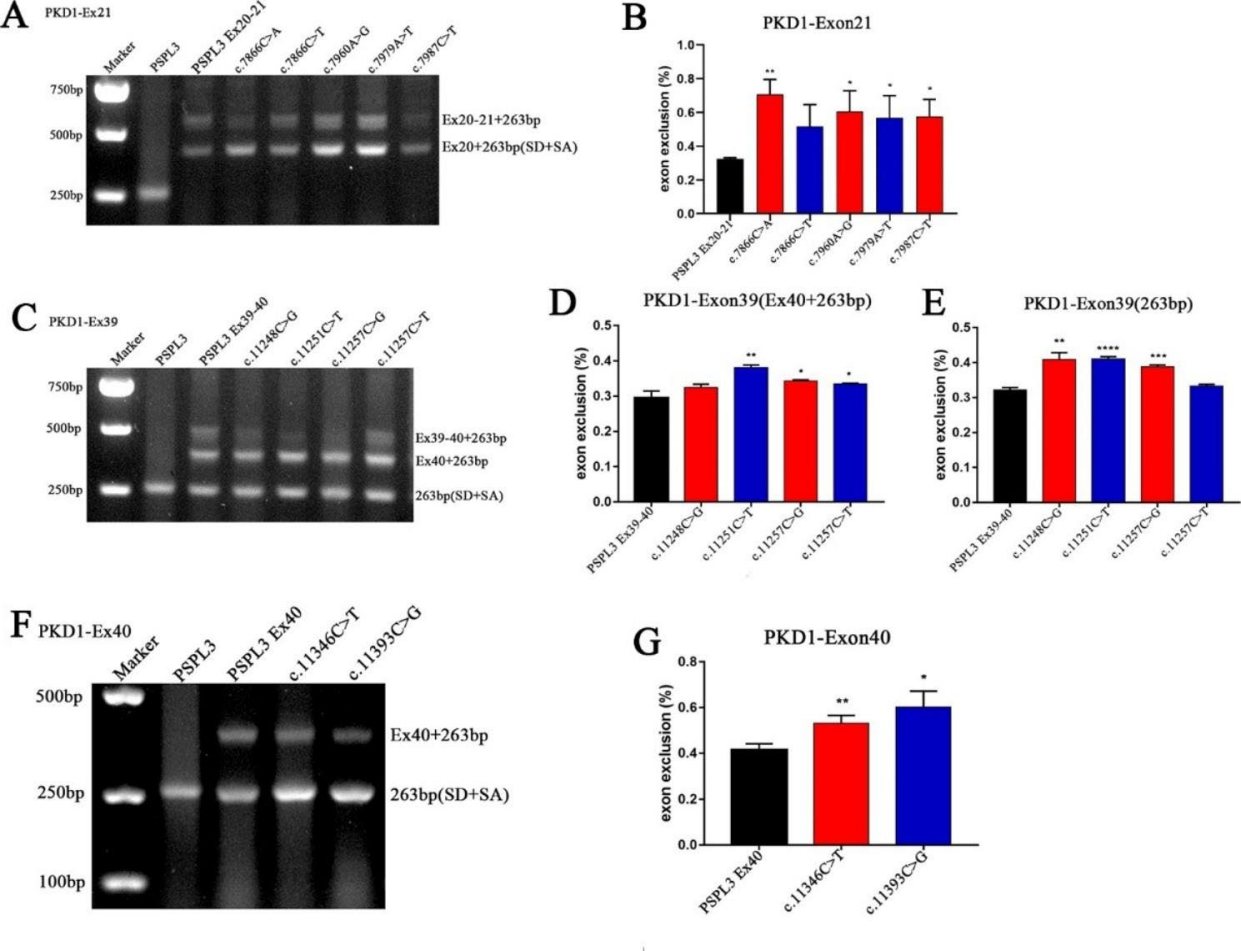
**Agarose gel electrophoresis and statistical analysis of RT-PCR products of *PKD1*** **gene**. (**A**) Lane 1: marker; lane 2: PSPL3 (263 bp); Lane 3: PSPL3 Ex20-21 (576 bp and 423 bp); Lane 4: c.7866C > A (576 bp and 423 bp); Lane 5: c.7866C > T (576 bp and 423 bp); Lane 6: c.7960A > G (576 bp and 423 bp); Lane 7: c.7979A > T (576 bp and 423 bp); Lane 8: c.7987C > T (576 bp and 423 bp). (**C**) Lane 1: marker; lane 2: PSPL3 (263 bp); Lane 3: PSPL3 Ex39-40 (518 bp, 405 bp and 263 bp); Lane 4: c.11248C > G (518 bp, 405 bp and 263 bp); Lane 5: c.11251C > T (518 bp, 405 bp and 263 bp); Lane 6: c.11257C > G (518 bp, 405 bp and 263 bp); Lane 7: c.11257C > T (518 bp, 405 bp and 263 bp). (**F**) Lane 1: marker; lane 2: PSPL3 (263 bp); Lane 3: PSPL3 Ex40 (405 bp and 263 bp); Lane 4: c.11346C > T (405 bp and 263 bp); Lane 5: c.11393C > G (405 bp and 263 bp). (**B, D, E, G**) Quantification of the splicing percentage was denoted by the percentage of exon exclusion (%) was calculated as (target band/ [lower band + (middle band) + upper band]) × 100. Error bars represent SEM (n = 3). *p < 0.05; **p < 0.01; ***p < 0.001; ****p < 0.0001, unpaired Student’s t-test.

The result of the minigene analysis in Hela and Human epithelial kidney 293T (HEK 293T) cells demonstrated that each WT lane produced two distinct segments of 576 bp and 423 bp, respectively (Fig. 2A). Direct sequencing revealed that the larger fragment contained exon 20 and exon 21 of *PKD1* and the two exons of pSPL3 (SD and SA), while the smaller amplicons included only exon 20, SD and SA. Similarly, the agarose gel electrophoresis of these variants (*PKD1*): c.7866C > A (p. Tyr2622X), (*PKD1*): c.7960A > G (p. Arg2654Gly), (*PKD1*): c.7979A > T (p. Asp2660Val) and (*PKD1*): c.7987C > T (p. Gln2663X) showed the presence of two fragments with the same size as the WT bands (Fig. 2A). Quantitative analysis proved that these SNVs altered weak splicing, resulting in the aberrant transcripts compared with WT plasmids (65.6% vs. 33.0%, P < 0.01; 53.1% vs. 33.0%, P < 0.05; 50.1% vs. 33.0%, P < 0.05; and 51.8% vs. 33.0%, P < 0.05) (Fig. 2B).

##### The variant (*PKD1*): c.7866C > T (p. Tyr2622Tyr) of exon 21 did not alter pre-mRNA splicing

The synonymous variant (*PKD1*): c.7866C > T (p. Tyr2622Tyr), located at nucleotide position 3 on exon 21, was demonstrated to reduce the score of the acceptor site of exon 21 from 0.67 to 0.52 by BDGP and was predicted to destroy three ESEs by HSF 3.1. Nonetheless, the software of MaxEntScan evaluated that this alteration could enhance the score of the acceptor site from -32.48 to -28.24. Finally, minigene analysis of the variant c.7866C > T resulted in two products that matched in size with those generated by the respective WT minigene. However, statistical analysis suggested no significant difference of exon 21-skipping transcript (45.3% vs. 33.0%, P = 0.0618), so it was concluded that the variant c.7866C > T did not alter mRNA splicing (Fig. 2B).

#### Exon 39

The missense variant (*PKD1*): c.11248C > G (p. Arg3750Gly) is located at the 22nd nucleotide upstream of the 3′ end of exon 39. The result of the HSF 3.1 analysis indicated that this alteration inactivated 9 potential ESE sites and generated 5 potential ESS sites. The nonsense variant (*PKD1*): c.11251C > T (p. Gln3751X), caused by the 18th nucleotide upstream of the 3′ end of exon 39, was anticipated to eliminate five ESEs and generate four new ESSs by bioinformatics analysis with HSF 3.1. These missense variants (*PKD1*): c.11257C > G (p. Arg3753Gly) and (*PKD1*): c.11257C > T (p. Arg3753Trp) were both located 13th nucleotide upstream of the 3′ end of exon 39. These two substitutions were predicted to gain 10 and 8 ESSs and disrupt 3 and 1 ESEs, respectively. Variant c.11257C > T is considered harmful by both MutationTaster, PolyPhen-2 and ClinPred, while it is arguable that the variant c.11257C > G is considered potentially benign by PolyPhen-2 and pathogenic by MutationTaster and ClinPred.

The control minigene comprising exon 39 and exon 40 was constructed and then transfected into HEK 293T and Hela cells to verify these four variants. The recombinant WT plasmid produces three types of transcription products (Fig. 2C): one is the normal spliced transcript including exon 39 and exon 40 of *PKD1* with a size of 518 bp, another was 405 bp containing exon 40, SD and SA, and the rest was proved to comprise only the 263 bp sequence of the pSPL3 vector.

The complementary DNA (cDNA) analysis of these four alterations also produced three types of transcripts (Fig. 2C) that were the same size as the WT minigene. Quantitative analysis of the PCR products revealed that the amount of 263 bp band generated by the minigene of variant c.11248C > G was increased (40.2% vs. 32.9%, P < 0.01) (Fig. 2D), while the rate of 405 bp fragment of the variant c.11257C > T in the total transcripts was statistically significant (33.77% vs. 27.7%, P < 0.05) (Fig. 2E). And the amounts of both 263 and 405 bp transcripts of variants c.11251C > T and c.11257C > G were observably increased (263 bp, 41.7% vs. 32.9%, P < 0.0001 and 39.3% vs. 32.9%, P < 0.001; 405 bp, 37.4% vs. 27.7%, P < 0.01 and 33.9% vs. 27.7%, P < 0.05) (Fig. 2D, E).

#### Exon 40

The synonymous variant (*PKD1*): c.11346C > T (p. Asp3782Asp) and nonsense variant (*PKD1*): c.11393C > G (p. Ser3798X) are both in the middle of exon 40. Bioinformatic analysis of BDGP demonstrated that the donor splice site of exon 40 was predicted to be 0.82, but the acceptor splice site was predicted to be 0.34, which was low and prone to splicing recognition disorder. Results of HSF 3.1 analysis indicated that the variant c.11346C > T and c.11393C > G inactivates 5 and 3 potential ESEs and generates 3 and 7 potential overlapping ESSs, respectively. Minigene assay in HEK 293T and Hela cells validated that the cDNA products of WT, variant c.11346C > T and c.11393C > G were identical in size, with two transcripts of 405 bp and 263 bp (Fig. 2F). Direct sequencing of all products showed that the larger amplicon was the exon 40-included transcript and the smaller amplicon comprised only the pSPL3 vector sequence. However, analysis of cDNA indicated that the amount of exon 40 skipping transcript of the variant c.11346C > T and c.11393C > G was both significantly higher than that of control plasmids (c.11346C > T, 53.1% vs. 38.2%, P < 0.01; c.11393C > G, 52.1% vs. 38.2%, P < 0.05) (Fig. 2G).

### Splicing outcome of variants in the *PKD2*

#### Nonsense variant (*PKD2*): c.1480G > T (p. Glu494X) induced skipping of exon 6 compared with the WT plasmid

Nonsense variant (*PKD2*): c.1480G > T (p. Glu494X), located at the internal of exon 6, was predicted that it not only eliminated 9 ESEs (TATTG**G**, ATTG**G**A (RESCUE-ESE), ATTG**G**A (EIE), TTG**G**AA (RESCUE-ESE), TTG**G**AA (EIE), TG**G**AAA (RESCUE-ESE), TG**G**AAA (EIE), G**G**AAAT, **G**AAATT) but also generated 1 ESS (TG**T**AAA) by HSF 3.1 (affected nucleotide is bold). To evaluate the effect of variant on pre-mRNA splicing, the minigenes containing this nucleotide substitution and WT were created and transfected into HEK 293T and HeLa cells separately.

**Fig. 3:**
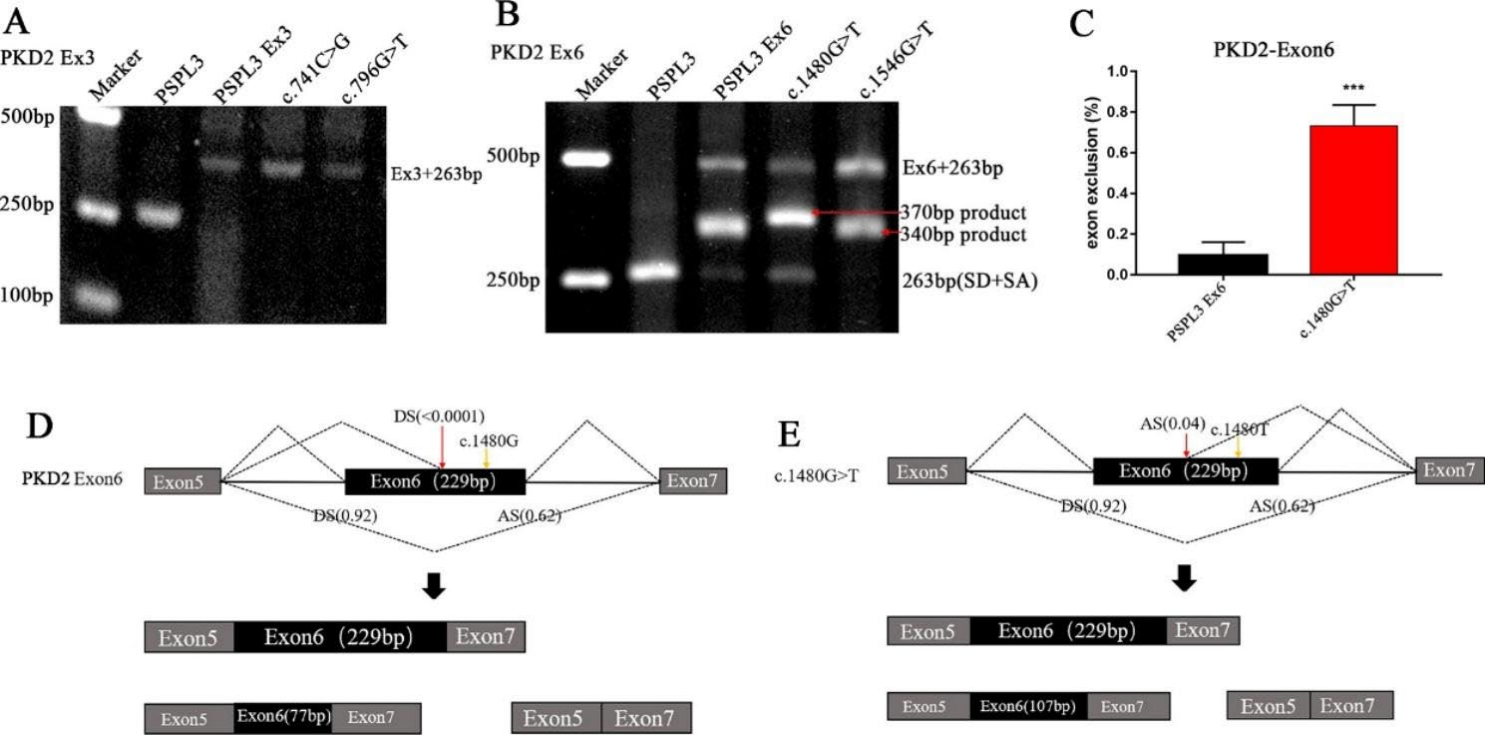
**Agarose gel electrophoresis and statistical analysis of RT-PCR products of *PKD2 *gene and functional characterization of c.1480G > T.** (**A**) Lane 1: marker; lane 2: PSPL3 (263 bp); Lane 3: PSPL3 Ex3 (397 bp); Lane 4: c.741C > G (397 bp); Lane 5: c.796G > T (397 bp). (**B**) Lane 1: marker; lane 2: PSPL3 (263 bp); Lane 3: PSPL3 Ex6 (492 bp, 340 bp, and 263 bp); Lane 4: c.1480G > T (492 bp, 370 bp, and 263 bp); Lane 5: c.1546G > T (492 bp, 340 bp, and 263 bp). (**C**) Quantification of the splicing percentage was denoted by the percentage of exon exclusion (%) was calculated as (target band/ [lower band + middle band + upper band]) × 100. Error bars represent SEM (n = 3). *p < 0.05; **p < 0.01; ***p < 0.001; ****p < 0.0001, unpaired Student’s t-test. (**D, E**) Structure model of the exon6 and the variant c.1480G > T in *PKD2* gene. Black and grey boxes represent PKD2 exons, and horizontal lines in between indicate intron segments. Their sizes are not proportional. Dotted lines show the splice sites used in each case

The RT-PCR products obtained from RNA of WT and variant minigenes were examined by agarose gel electrophoresis. Three different electrophoresis bands were detected in WT minigene: one of the bands with a size of 492 bp corresponded to the correctly splice exon 6, one product (340 bp) indicated alternative splicing, and another 263 bp transcript contains only the SD and SA of pSPL3 (Fig. 3B). Sequence analysis confirmed that the 340 bp product contained an incomplete exon 6 lacking 152 bp from the 5′ end. Miraculously, the minigene analysis of variant c.1480G > T revealed a new production (370 bp) which included a 122 bp fragment of exon 6 deletion from the 3′ end and two exons of pSPL3 (Fig. 3B). Furthermore, quantitative analysis suggested that there was a significant increase of the exon 6-skipping transcript of c.1480G > T compared with WT plasmids (Fig. 3C).

#### Nonsense variants (*PKD2*): c.741C > G (p. Tyr247*) and (*PKD2*): c.796G > T (p. Glu266*) in exon3 and missense variant (*PKD2*): c.1546G > T (p. Val516Leu) in exon 6 did not alter pre-mRNA splicing

Nonsense variants (*PKD2*): c.741C > G (p. Tyr247*) and (*PKD2*): c.796G > T (p. Glu266*) in the middle of exon 3 were predicted to result in ESEs/ ESSs imbalance (Table 1). However, analysis of minigene confirmed that they did not alter exon splicing (Fig. 3A). Missense variant (*PKD2*): c.1546G > T (p. Val516Leu), located at third nucleotide upstream of the 3 ‘end of exon 6, was forecasted to have a marginal increase in the score of 3′ splice site from 0.62 to 0.68 by BDGP, while a slight decrease the score from 7.23 to 7.0 3 by MaxEntScan. Besides, it could generate 6 ESSs and break 2 ESEs by HSF 3.1. Nevertheless, the RT-PCR products of the minigene containing the variant c.1546G > T matched in size with the corresponding WT minigene (Fig. 3B). This was further confirmed by sequencing analysis, which is consistent with previous predictive results of Polyphen-2 on the protein level. Therefore, these variants, c.741C > G, c.796G > T, and c.1546G > T, were considered to have no influence on pre-mRNA splicing.

## Discussion

Alteration of the pre-mRNA splicing has long been considered a potential cause of rare genetic diseases [25, 26]. In addition to classifying exon variants as missense, synonymous, or nonsense alterations at the DNA level, the pathogenicity of single nucleotide substitution should also be evaluated at the RNA level. However, RNA analysis is generally difficult due to the activation of nonsense-mediated mRNA pathway decay or the inability to obtain RNA samples from infected individuals. Currently, functional splicing detection based on minigene analysis is a better solution. And in Dr. Hansen’s study, the minigene analysis suggested 100% concordance with RT-PCR analysis of RNA from blood samples/lymphoblastoid cell lines [27]. Thus minigene assay has proven to be an effective, reliable and relatively simple tool for the functional analysis of potential splicing alterations [28], which has been widely verified in our previous studies [17, 29]. Herein, we hypothesize that some SNVs may play a pathogenic role through the alteration of pre-mRNA splicing and the minigene assay was performed for 19 variants of the *PKD1* and *PKD2* genes selected by bioinformatics tools. Eventually, 11 of them were demonstrated to result in exon skipping.

Splicing signals that regulate the splicing process include ESEs and ESSs, which can coordinate the correct splicing of exons by recruiting different protein factors to promote or inhibit recognition of surrounding splicing sites [26]. In the *PKD1* gene, transcriptional PCR analysis of these variants (c.7866C > A, c.7960A > G, c.7979A > T, c.7987C > T, c.11346C > T and c.11393C > G) have shown that they affect normal pre-mRNA splicing by causing a significant imbalance in the ESE/ESS ratios. It is important to note that the mechanism by which the variant c.7866C > A affected splicing is complex, and it may also alter the recognition ability of acceptor site because it was located at the 5′ end of the exon 21. Eventually, these variants c.7866C > A, c.7960A > G, c.7979A > T and c.7987C > T, to varying degrees, prevented the inclusion of exon 21, resulting in an in-frame deletion (codon 2622–2672) in which mutant proteins would lose part of extracellular N-terminal domain of PC1 [30].

BDGP analysis of exon 39 in *PKD1* showed that the donor site score was 0.29 and the acceptor site score was 0.88, suggesting that this exon had a high probability of aberrant splicing. Meanwhile, bioinformatics results indicated that these four variants (c.11248C > G, c.11251C > T, c.11257C > T, and c.11257C > G) in exon 39 also significantly changed the ESE/ESS ratios. Minigene analysis demonstrated that these alterations could result in skipping of exon 39 and 40 together or exon 39 alone. Functional analysis from the protein level, the skipping of exon 39 alone generated a truncated protein (p. Ser3720Thr fs*58), while simultaneous skipping of exon 39 and 40 contributed to an in-frame deletions (codon 3720–3804), affecting the TOP domain of PC1 [31]. Both these mutated proteins prevented PC1 from performing its normal physiological function in the kidney by affecting the connection between PC1 and PC2, thus promoting the cyst formation. Surprisingly, combined with Dr Claverie-martin’s results, all the different variants at nucleotide 11257 in exon 39 could cause splicing abnormalities [18]. This may imply that the region of this nucleotide site is highly conserved, which may be highly related to the ESEs/ESSs imbalance or the RNA secondary structure variation, whereas its specific function needs further study.

Generally, the synonymous variants don’t modify the amino acid sequence and affect protein function because of the genetic codon degeneracy, so it will be listed as “benign” or “possibly benign” according to the American Medical Genetics and Genomics (ACMG) guidelines [32]. However, we discovered that the synonymous variant c.11346C > T could disrupt 5 ESEs and generate 3 ESSs by bioinformatics software. The minigene splicing assay revealed that the synonymous variant c.11346C > T and nonsense variant c.11393C > G resulted in exon 40 skipping of *PKD1* with a subsequent frameshift at amino acid 3757 and premature truncation following the addition of 21 amino acid (p. Ala3757Gly fs*22). Studies have shown that the key site of the PC1/PC2 complex connection is located at amino acids *PKD1*^3049–4169^ and *PKD2*^185–723^ [31]. Therefore, it may be the pathogenic mechanism of these two alterations, where the truncated PC1 affects the binding with PC2, causing the loss of function of this protein in the kidney.

The analysis of the minigene gene containing *PKD2* wild-type exon 6 revealed the presence of alternative splicing, which may be associated with a very weak acceptor site (score < 0.001 by BDGP) within the exon 6 sequence, resulting in a deletion of 152 bp at the 5′ end (Fig. 3D). Remarkably, the nonsense variant c.1480G > T (p. Glu494X) generated an abnormal transcript with a 122 bp deletion of the 3′ end by the activation of a cryptic donor site (cagcctGTgagatt, score: 0.04 by BDGP) (Fig. 3E) located 54 bp upstream from the nucleotide change. In addition, the product of complete exon 6 skipping was also transcribed by the recombinant minigene of this alteration, which could be due to the elimination of ESEs within exon 6.

As mentioned above, PC2 is a calcium-activated non-selective cation channel that plays a regulatory role in the kidney by forming a protein complex with PC1 through C-terminal binding. In other studies, the missense variant (*PKD2*): c.1532A > T (p. Asp511Val) was also demonstrated to activate a mysterious donor site inside exon 6, forming an aberrant splicing amplicon identical to the variant c.1480G > T [19]. Peter Koulen and his colleagues have identified that the missense variant c.1532A > T can result in a complete loss of PC2 channel activity [33]. By contrast, a truncated PC2 polypeptide produced by the nonsense substitution (p. Leu703X) retains channel activity. However, it is no longer calcium-activated or voltage-dependent, nor does it release calcium from intracellular stores [33]. Furthermore, the *PKD2* transcript without exon 7, a product of alternative splicing, generated a significantly altered protein alteration (p. Ser518Phe fs*394) that affects the C-terminus and prevents PC2 from binding to PC1 to form a protein complex [34]. Therefore, these mRNAs generated by variant c.1480G > T can be translated into three truncated proteins (p. Glu494X corresponds to the transcript containing the variant c.1480G > T; p. Cys476Ser fs*9 corresponds to the splicing product with a 122 bp deletion at the 3′ end; and p. Arg440Ser fs*5 corresponds to the mRNA of a complete skipping of exon 6), which may affect the function of proteins through different pathways, conducing to the formation of kidney cysts. However, the specific pathogenic mechanism of the variant c.1480G > T is still unclear and further studies are needed to prove it.

Significantly, results of minigene assay confirmed that these single nucleotide substitutions mentioned above not only produced the fragments with complete or incomplete skipping of the exon, but also generated some correct splicing transcripts. Increasing evidence suggests that PC1 and PC2 inhibit cyst formation in a dose-dependent manner, and the cystogenesis occurs when the concentration of abnormal PC1 or PC2 falls below a certain threshold [35, 36]. This partly explains the fact that people who have both correct and abnormal splicing still exhibit ADPKD phenotype.

Moreover, although these variants distributing in *PKD1* (c.7866C > T) and *PKD2* (c.741C > G, c.796G > T and c.1546G > T) were predicted to affect the recognition strength of splicing site by BDGP or influence surrounding ESEs and ESSs sequences by HSF 3.1, minigenes assays demonstrated that they did not contribute to abnormal exon skipping. Interestingly, unlike the “benign” or “polymorphism” predicted by the PolyPhen-2 or the MutationTaster, c.7960A > G and c.11257C > G were actually “detrimental” variants. In contrast, results of the minigene and ClinPred were consistent in the predictions of missense variants. Taken together, these indicated the limitations of the software prediction. Notably, it is difficult for the minigene analysis to fully reflect the situation in the body because of the complex regulatory mechanisms. In addition, the secondary structure of mRNA may also affect translation results, and the expression of splicing factors may be different in different cell lines. Analysis of kidney RNA samples from patients is recommended before drawing any conclusions about the pathogenicity of variants.

## Conclusion

In summary, we have performed a comprehensive analysis of exonic variants in *PKD1* and *PKD2* using bioinformatics tools and minigene assay. Eleven variants (c.7866C > A, c.7960A > G, c.7979A > T, c.7987C > T, c.11248C > G, c.11251 C > T, c.11257C > G, c.11257C > T, c.11346C > T, and c.11393C > G distributed in *PKD1* and c.1480G > T located in *PKD2*) that were previously described as missense, synonymous, or nonsense variants in ADPKD patients were confirmed to be pathogenic by affecting pre-mRNA splicing. This study emphasized the significance of assessing the effect of SNVs at the mRNA level in ADPKD and validated that minigene analysis could be a valuable tool, especially when RNA or kidney specimens are not available. Moreover, our results help to validate previously unpredictable splicing regulatory elements to better understand the splicing regulation mechanisms of pre-mRNA in *PKD1* and *PKD2*.

## Materials and methods

### Variant nomenclature

DNA variant numbering is based on the cDNA sequence for *PKD1* (Ensembl: ENST00000262304.9) and *PKD2* (Ensembl: ENST00000237596.7). The nomenclature of variants followed the guidelines of the Human Genome Variation Society (http://varnomen.hgvs.org), with c.1 representing the first position of the translation initiation codon.

### Bioinformatics predictions and screening criteria

All SNVs in *PKD1* and *PKD2* were selected from the HGMD (professional, accessed February 2021), ADPKD Mutation Database (accessed February 2021). Different complementary online bioinformatics software was employed to determine the possible effects of alterations on pre-mRNA processing. The BDGP (http://www.fruitfly.org/) and MaxEntScan (http://hollywood.mit.edu/burgelab/maxent/Xmaxent.html) were performed to analyze the potential effects of variant on the classic 5′ donor or 3′ acceptor site and predict the generation and/or activation of new sites. The online software Human Splice Finder 3.1 (https://www.genomnis.com/access-hsf) was used to investigate the existence of potential splicing regulatory sequences (ESEs and ESSs) and to determine the possible influence of substitutions on splicing regulatory sequences. To evaluate the potential effects of missense variants, the MutationTaster (https://www.mutationtaster.org/), Polymorphism Phenotyping v2 (http://genetics.bwh.harvard.edu/pph2/) and ClinPred (https://sites.google.com/site/clinpred/) were further applied. The role of SnapGene software is to perform predictive analysis of reading frame changes and following protein defect for experimentally validated variants that alter exon splicing.

In this study, the screening criteria for the single nucleotide substitutions of the *PKD1* and *PKD2* were as follows. Firstly, the satisfying exons with BDGP score below 0.5 were selected to continue the analyses. Then, bioinformatics software (BDGP, MaxEntScan and HSF 3.1) was used for all SNVs in these exons to assess the effects on exon splicing sites and exon splicing regulatory elements (the total number of ESE disruption and ESS generation is more than 8). Besides, potential splicing variants within 3 bases closed to the 5′ or 3′ end of the exon were also included.

### Minigene constructions

**Fig. 4:**
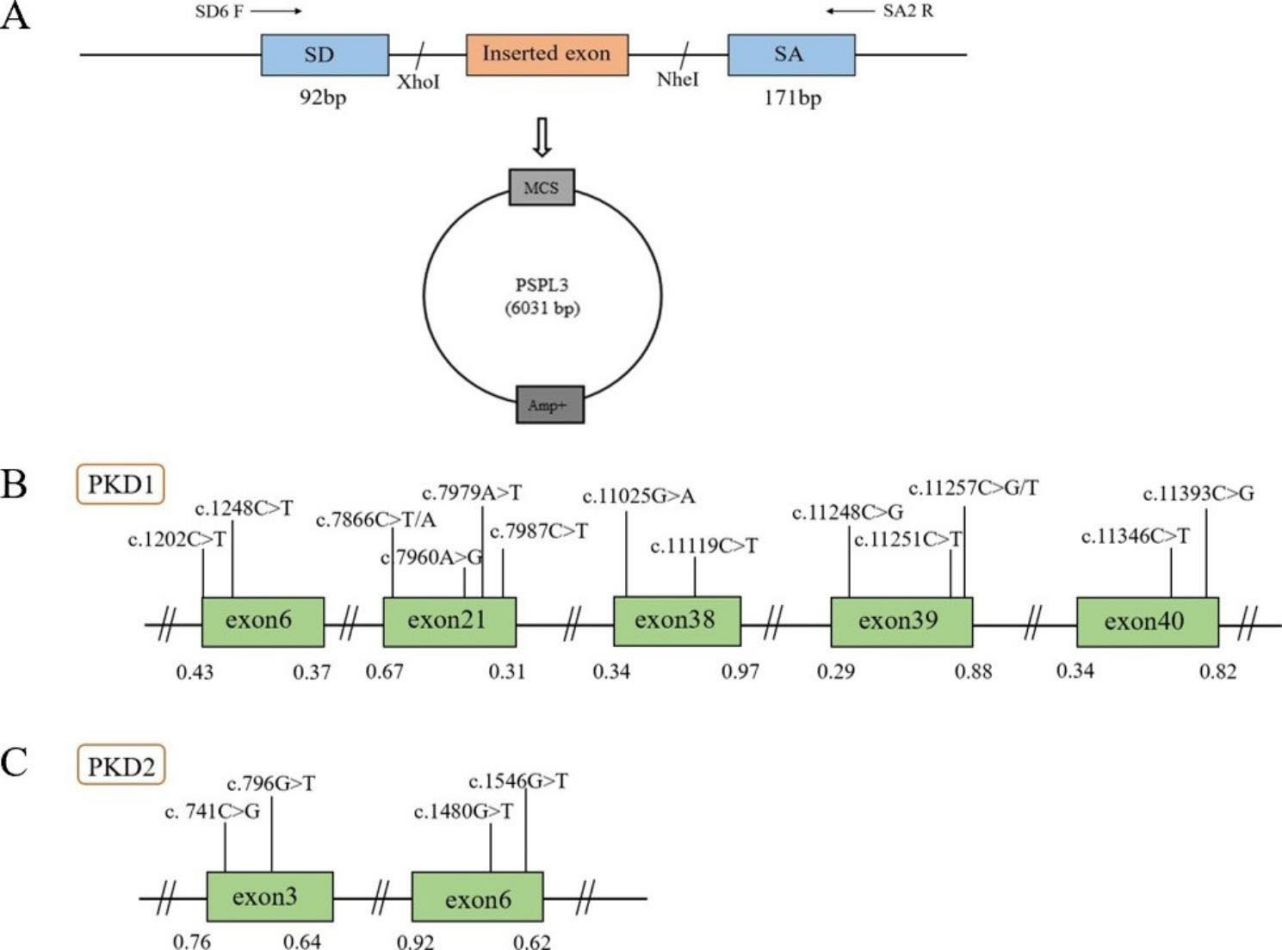
**The schematic diagram of minigene based on the pSPL3 exon trapping vector and position of 19 presumed exonic variants.** (**A**) The wild-type and mutant fragments of the target exon were connected to pSPL3 vector via XhoI and NheI cloning sites of pSPL3 vector, respectively, to form wild-type and mutant pSPL3 plasmids. (**B, C**) Position of candidate variants in *PKD1* and *PKD2* gene. Green boxes and black lines between them represent the coding exons and introns sequences, respectively. Their sizes are not proportional. The BDGP scores of donor and acceptor splice sites are represented in decimal

In order to investigate the effect of the candidate single nucleotide alteration on the splicing process, a minigene splicing assay based on the pSPL3 exon capture vector was used for in vitro analysis, and minigene constructions have been described as previously reported (Fig. 4) [17]. Specific primers linking the XhoI and NheI restriction enzyme sites (XhoI: TGGAGC^TCGAG; NheI: AATTTG^CTAGC) were used to amplify the exons in which the screened variant resides and 50–200 bp of their intronic flanking regions. Then they were cloned into the splicing vector pSPL3 to form WT plasmid. Primers were designed for each target fragment using Primer X5 (Supplementary Table S1). Selected substitutions were introduced into WT plasmid by site-directed mutagenesis using GeneArt Site-Directed Mutagenesis PLUS System (Thermo Fisher Scientific) as instructed by the manufacturer and mutagenesis primers are listed in Supplementary Table S2. All constructed vectors were transformed into Escherichia coli DH5α‐competent cells (TaKaRa) for amplification. All constructs were verified by sanger sequencing (as shown in Supplementary Figure S1).

### Transient transfection and RNA extraction

HEK 293T and HeLa cells both were purchased from the American Type Culture Collection (ATCC, USA). These two kinds of cells were grown in DMEM medium (Procell, China) supplementing with 10% fetal bovine serum (Procell, China), penicillin (100 U/ L, Procell), and streptomycin (100 mg/L, Procell). Cell cultures were incubated at 37 °C and 5% CO2 in a humidified incubator. One day before transfection, both cells were transferred to a 6-well culture plate, where they grew to approximately 70–80% fusion in an antibiotic-free medium. Each group of plasmids (empty pSPL3- control (EV), pSPL3‐WT and pSPL3‐Mutation (MUT), 2 µg each) were transfected to HEK 293T and Hela cells using OPTI-MEM® IMedium and Lipofectamine 3000 (Invitrogen, Carlsbad, CA, USA) according to the manufacturer’s instructions. As previously reported, an aliquot of each culture was co-cultured with puromycin to prevent nonsense-mediated RNA decay [18]. After 48 h of incubation, cells were harvested and total RNA was extracted using the TRIzol reagent (Invitrogen).

### Analysis of minigenes

First-strand cDNA was synthesized from 2 µg of total RNA through random‐primed reverse transcription with Superscript II Reverse Transcriptase (Invitrogen Corporation). The resulting cDNA was amplified by PCR using vector-specific primers: SD6 (the forward primer: 5′-TCTGAGTCACCTGGACAACC-3′) and SA2 (the reverse primer: 5′-ATCTCAGTGGTATTTGTGAGC-3′).

The PCR amplification reaction was performed as follows: in 50 µl volume, 2 µl of cDNA, 25 µL of 2 × PrimeSTAR (Premix) (TaKaRa, Japan), 1 µM of each primer in a 9700 (Applied Biosystem, Foster City, CA, USA) thermal cycler. Thermal conditions are 29 cycles of 98 ℃ for 30 s, 58 ℃ for 30 s, elongation at 72 ℃ for 90 s, and finally an extension step at 72 ℃ for 10 min. The PCR products were separated by electrophoresis on a 1.5% agarose gel, and each band intensity was quantified by the software Image J. The target DNA bands were purified using a Gel Extraction Kit (CWBIO), and then the transcripts were analyzed by DNA sequencing (as shown in Supplementary Figure S2, S3, S4, and S5). If a splicing pattern different from WT minigene was observed in both cell lines, the variation was considered to result in a splicing defect.

### Statistical analysis

The percentage of exon exclusion (%) was calculated as (target band/ [lower band + (middle band) + upper band]) × 100. Statistical analysis was performed using GraphPad Prism (Version 6.02, GraphPad Software, USA). The results were analyzed using the two-tailed Student’s t-test or one-way ANOVA test. Error bars represent SEM (n = 3). P < 0.05 was considered statistically significant.

### Electronic supplementary material

Below is the link to the electronic supplementary material.

Supplementary Material 1

Supplementary Material 2

Supplementary Material 3

Supplementary Material 4

Supplementary Material 5

Supplementary Material 6

Supplementary Material 7

Supplementary Material 8

Supplementary Material 9

## Data Availability

All data generated or analyzed during this study are included in the article/Supplementary Material, further inquiries can be directed to the corresponding authors. The variants collected in this study are available in HGMD (Professional, http://www.hgmd.cf.ac.uk/ac/validate.php/) and ADPKD Mutation Database (https://pkdb.mayo.edu/). The websites for bioinformatics analysis of variants are shown as follow: The BDGP (http://www.fruitfly.org/). MaxEntScan (http://hollywood.mit.edu/burgelab/maxent/Xmaxent.html). Human Splice Finder 3.1 (https://www.genomnis.com/access-hsf). MutationTaster (https://www.mutationtaster.org/), Polymorphism Phenotyping v2 (http://genetics.bwh.harvard.edu/pph2/). ClinPred (https://sites.google.com/site/clinpred/).

## **Abbreviations**

pre-mRNA: Precursor messenger RNA
mRNA: Messenger RNA
SNVs: Single nucleotide variants
DS: Donor site
AS: Acceptor site
BPS: Branch point sequence
PY: Polypyrimidine tract
ESEs/ISEs: Exonic/intronic splicing enhancers
ESSs/ISSs: Exonic/intronic splicing silencers
SR: Serine/arginine-rich protein family
hnRNPs: Heterologous nuclear ribonucleoproteins
ADPKD: Autosomal dominant polycystic kidney disease
PC1: Polycystic protein-1
PC2: Polycystic protein-2
HGMD: Human Gene Mutation Database
BDGP: Berkeley Drosophila Genome Project
WT: Wild-type
HEK 293T: Human epithelial kidney 293T
cDNA: Complementary DNA
