# Supplementary figures and images for: Identified eleven exon variants in PKD1 and PKD2 genes that altered RNA splicing by minigene assay
Source: BMC Genomics. 2023 Jul 19;24:407. doi: 10.1186/s12864-023-09444-9 (PMC10354997; doi:10.1186/s12864-023-09444-9)

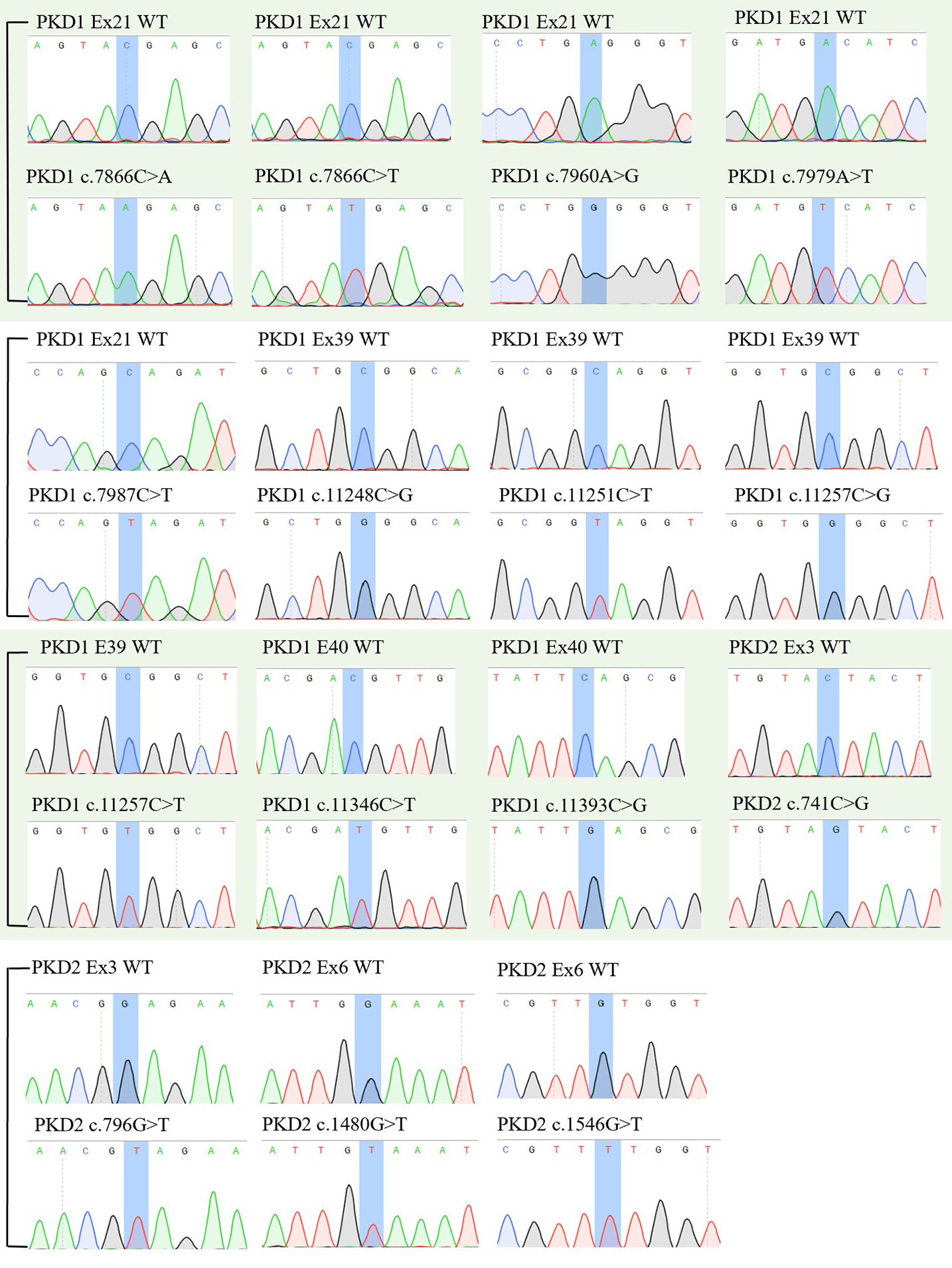

Supplement: Supplementary file 1 — Supplementary Material 1 [file 12864_2023_9444_MOESM1_ESM.tif]

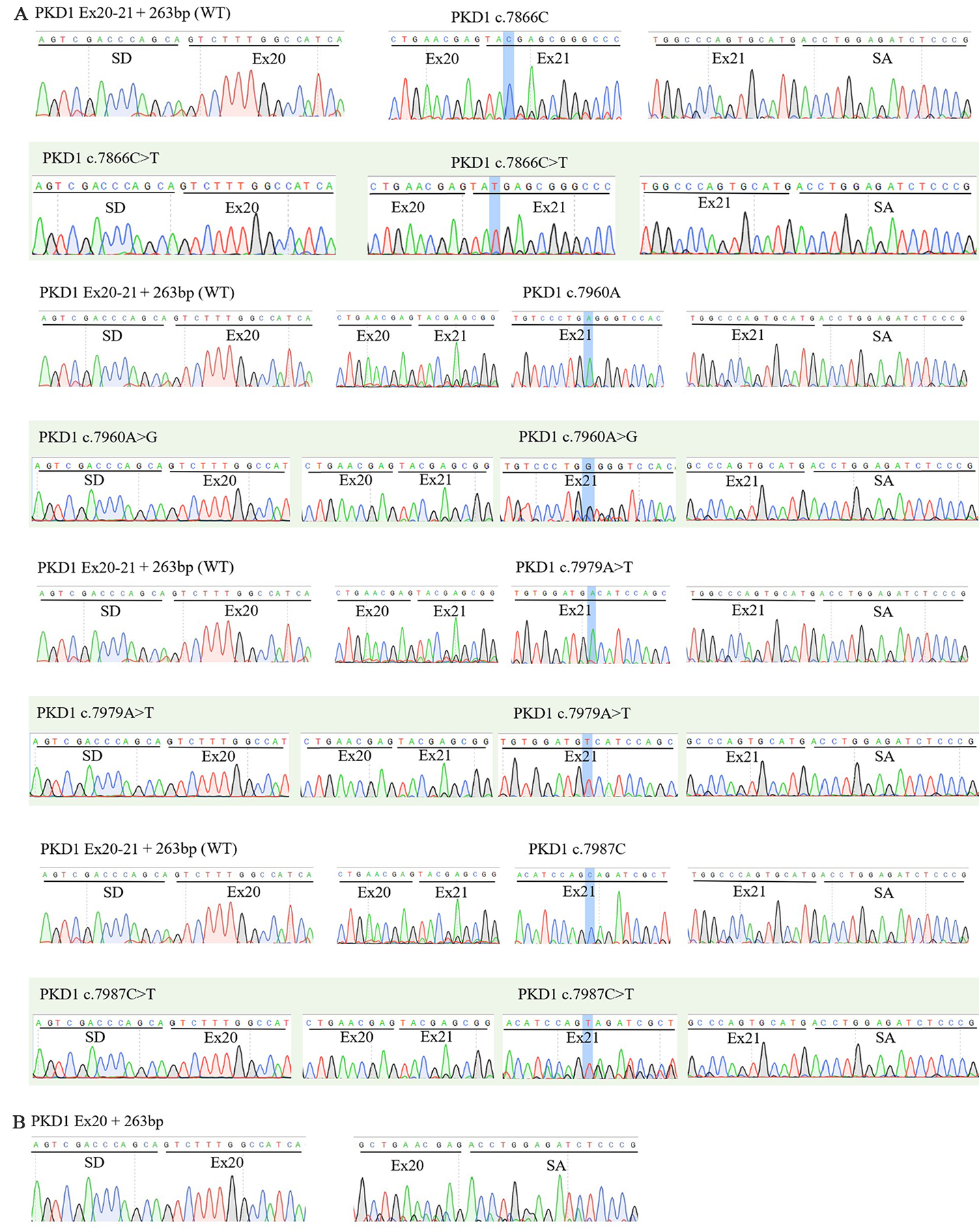

Supplement: Supplementary file 2 — Supplementary Material 2 [file 12864_2023_9444_MOESM2_ESM.tif]

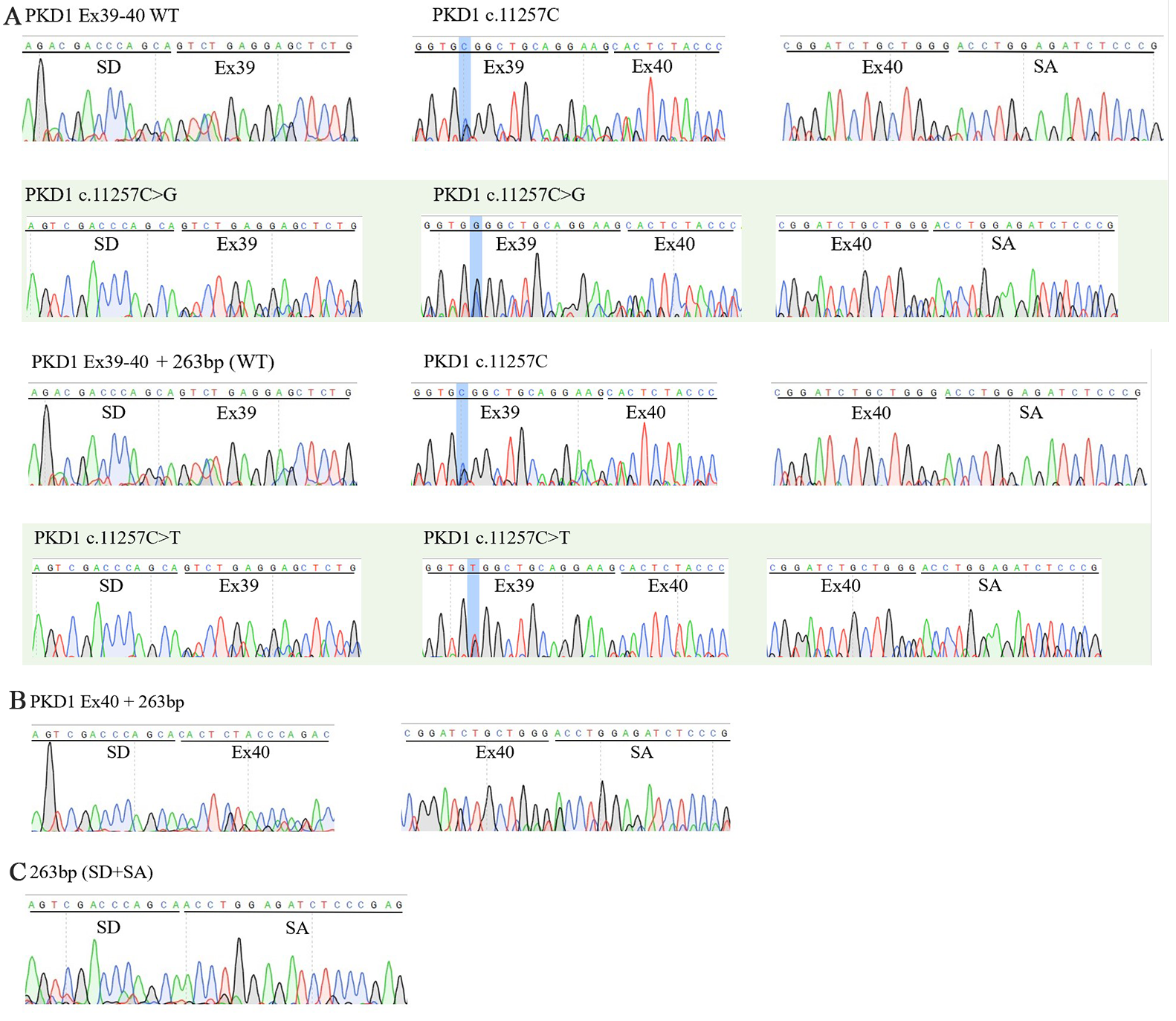

Supplement: Supplementary file 3 — Supplementary Material 3 [file 12864_2023_9444_MOESM3_ESM.tif]

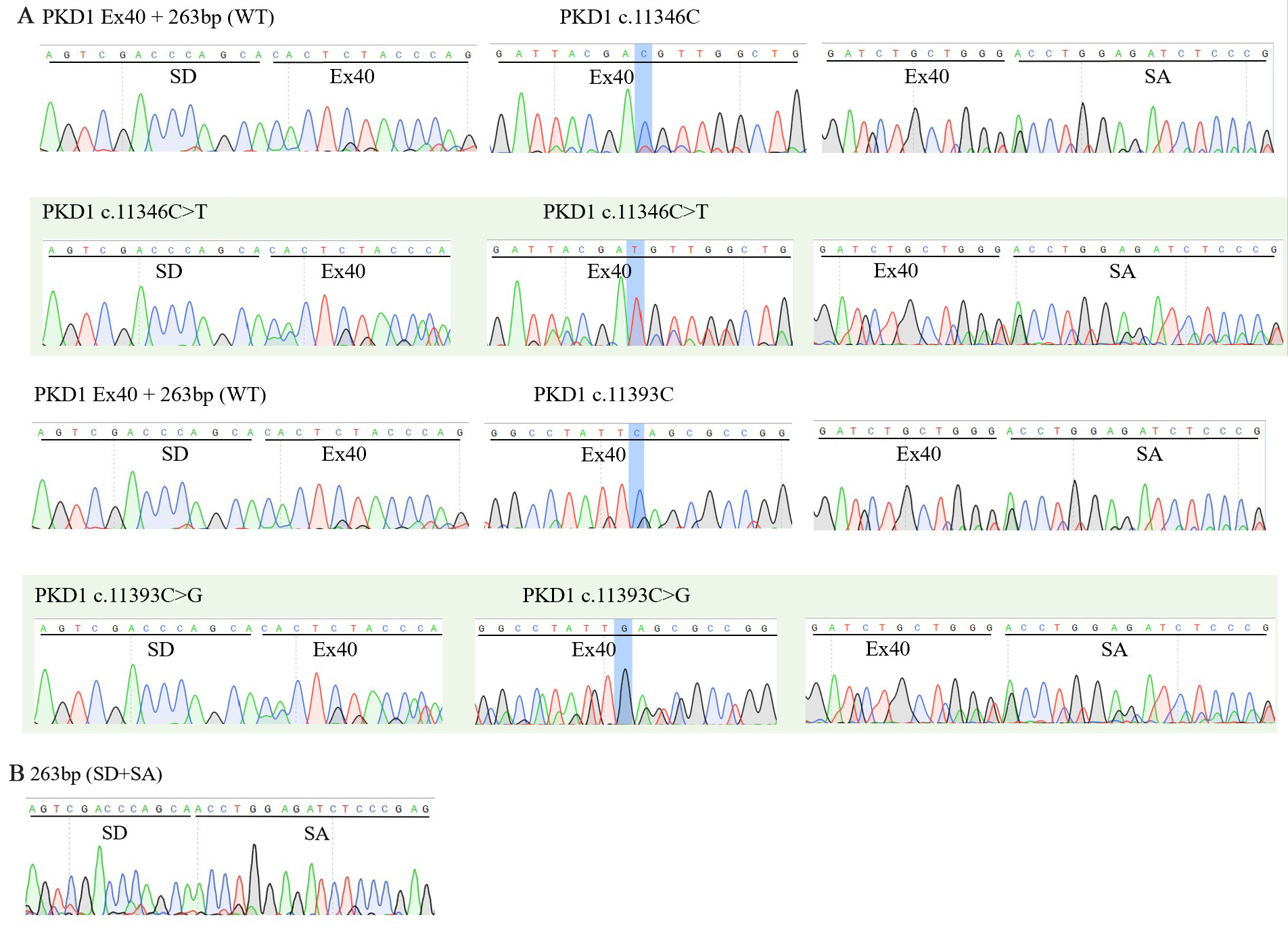

Supplement: Supplementary file 4 — Supplementary Material 4 [file 12864_2023_9444_MOESM4_ESM.tif]

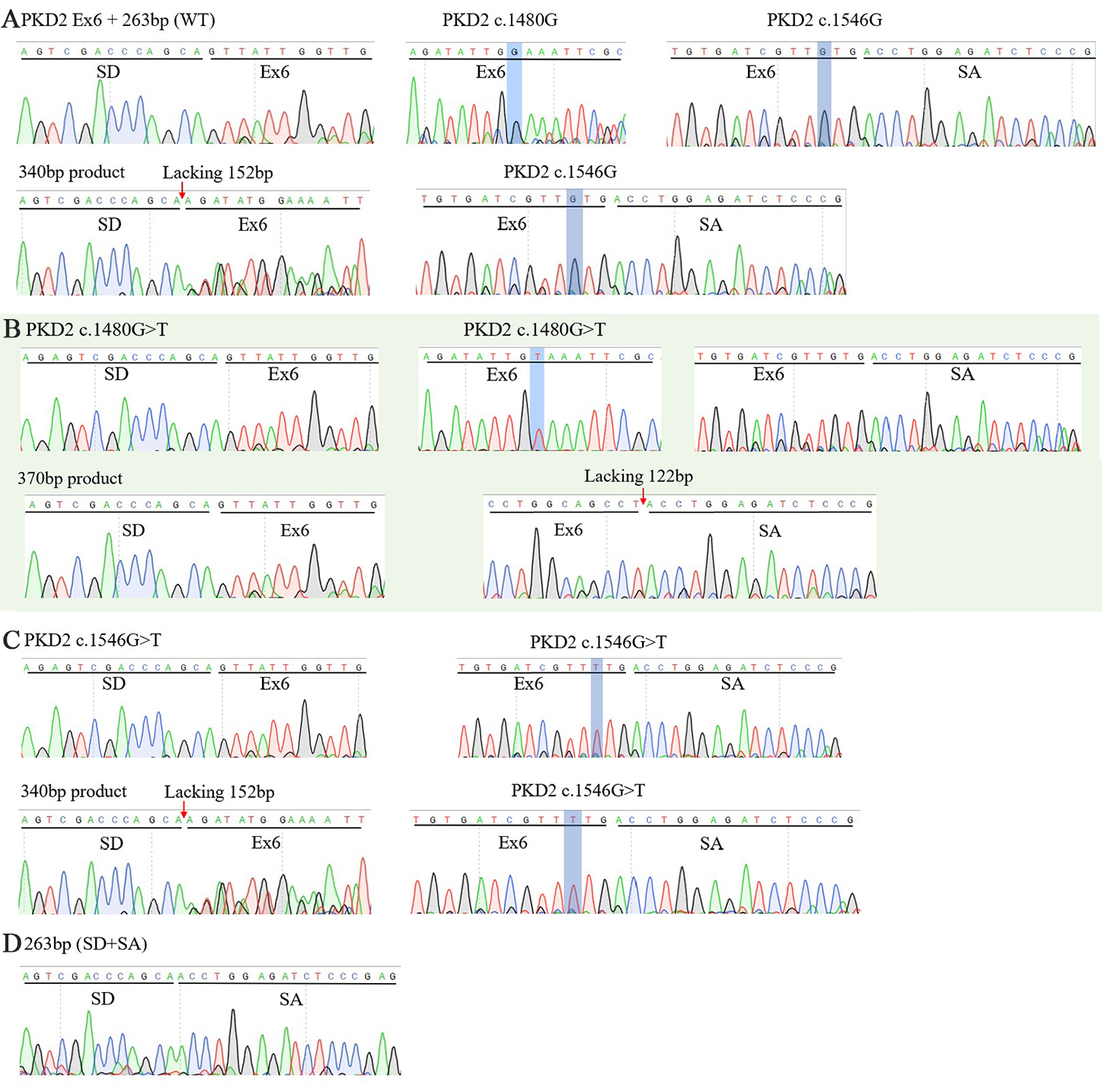

Supplement: Supplementary file 5 — Supplementary Material 5 [file 12864_2023_9444_MOESM5_ESM.tif]

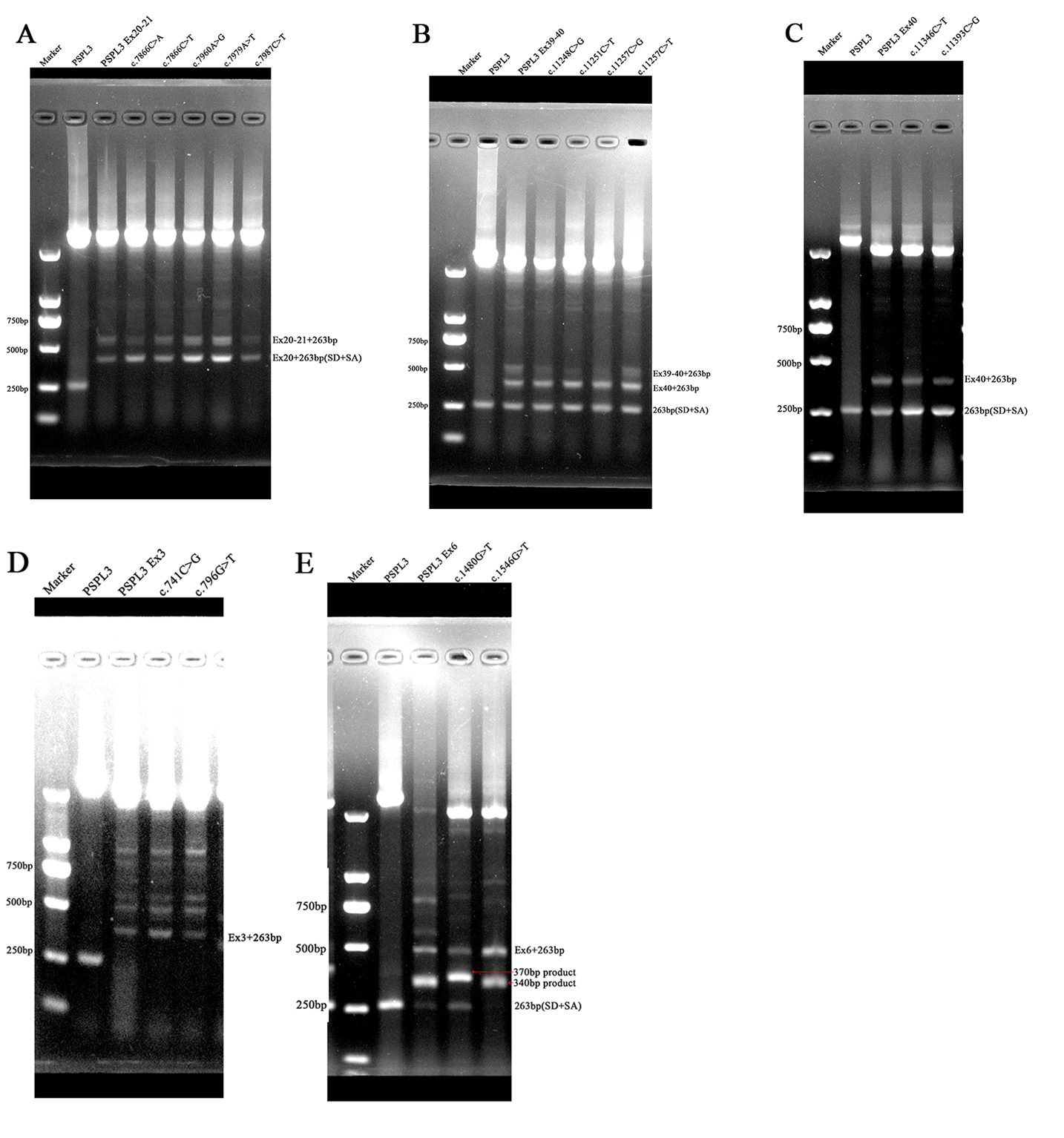

Supplement: Supplementary file 6 — Supplementary Material 6 [file 12864_2023_9444_MOESM6_ESM.tif]
