# Supplementary material for: Identified eleven exon variants in PKD1 and PKD2 genes that altered RNA splicing by minigene assay
Source: BMC Genomics. 2023 Jul 19;24:407. doi: 10.1186/s12864-023-09444-9 (PMC10354997; doi:10.1186/s12864-023-09444-9)
Supplement: Supplementary file 7 — Supplementary Material 7 [file 12864_2023_9444_MOESM7_ESM.docx]

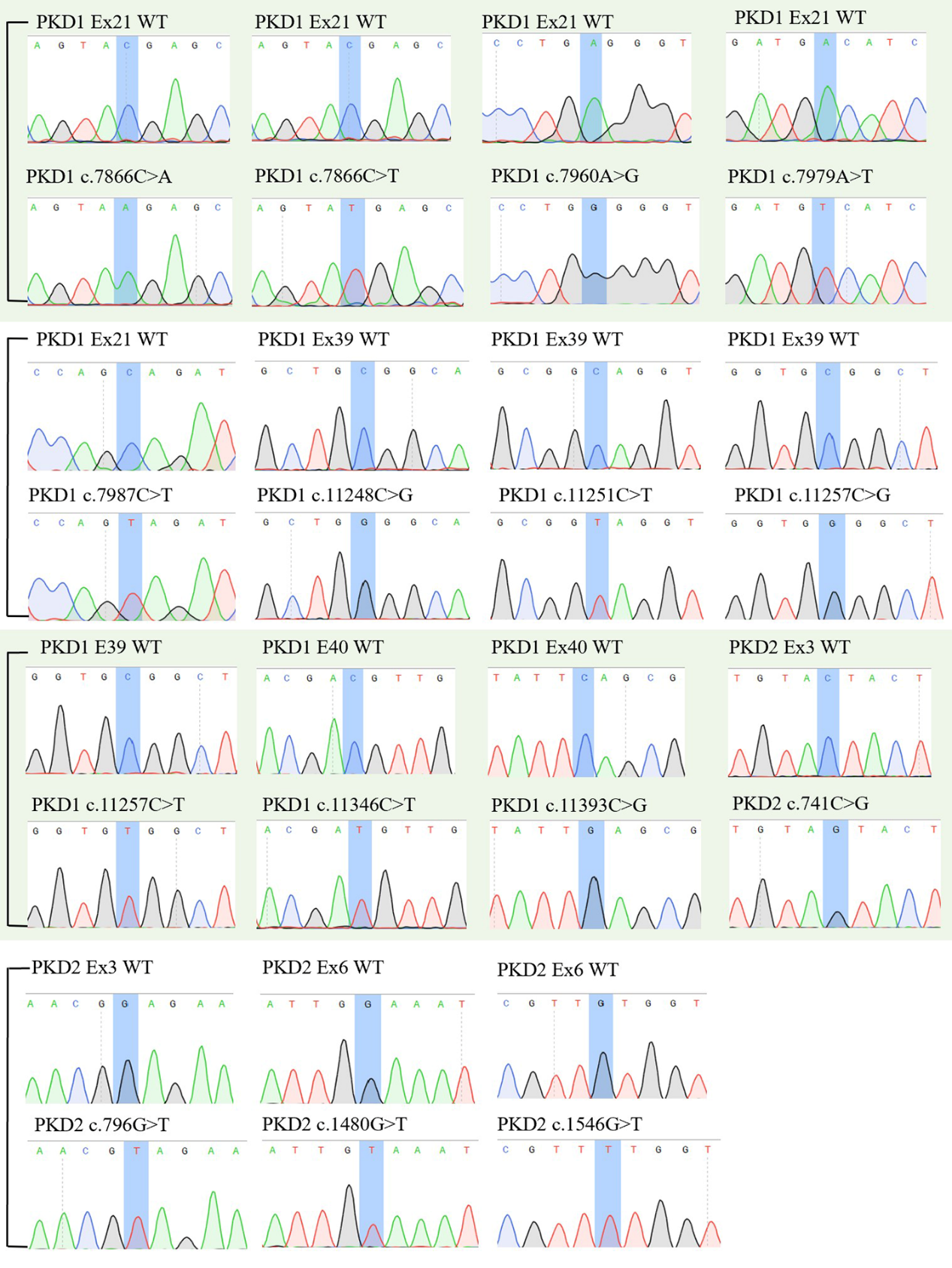


**Supplementary Figure S1. The sanger sequencing results of all constructed recombinant plasmids.** The blue boxes indicate the mutation sites.

**
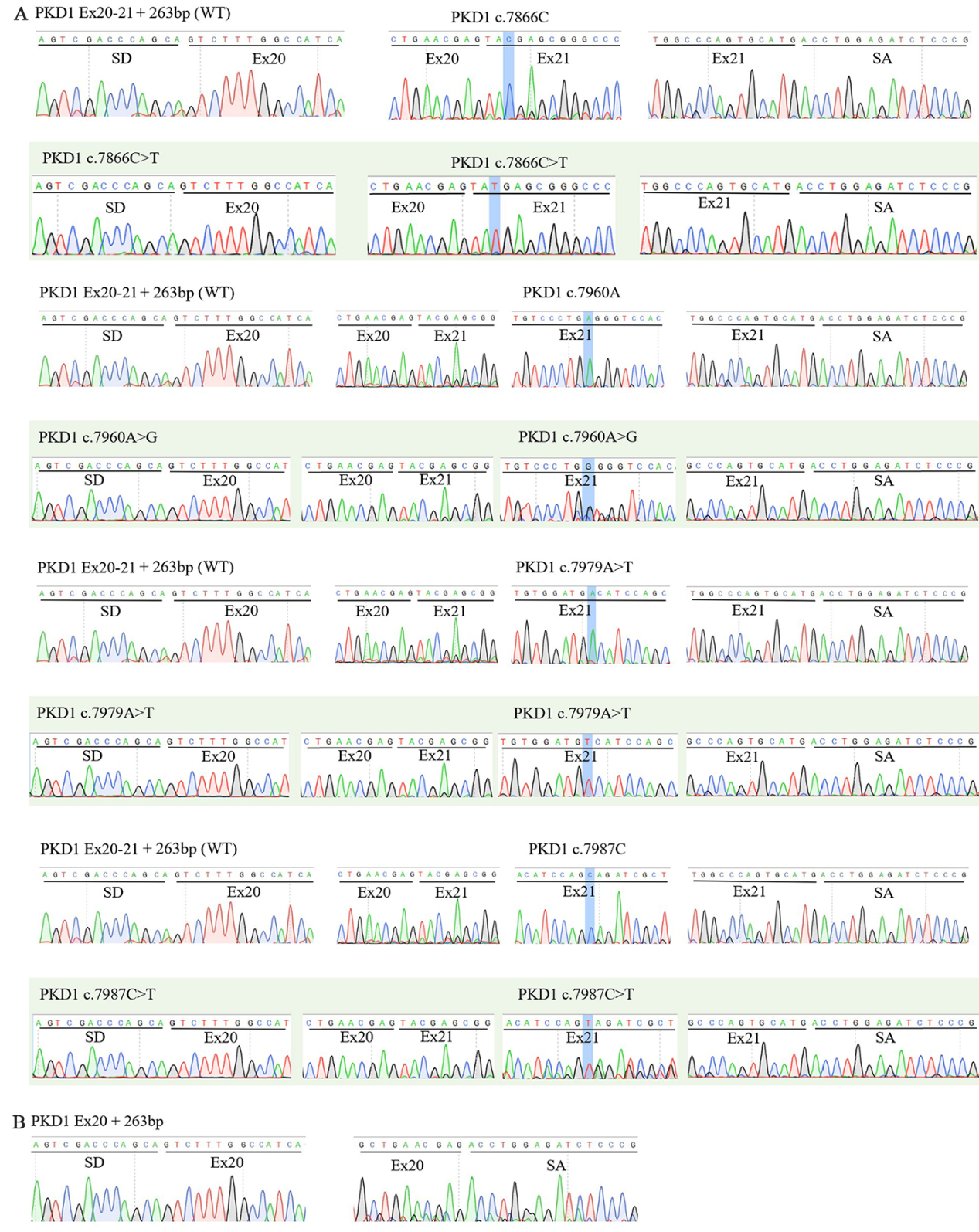
**

**Supplementary Figure S2. Sequencing diagrams of transcripts of variants on exon 21 of *PKD1.*** (A) The fragment contained the exon 20 and exon 21, flanked by two exons of the pSPL3 vector (SD and SA, 263bp). (B) The segment included only exon 20 and pSPL3 vector sequences. The blue boxes indicate the mutation sites. The sequencing results were continuous, but the presentation was disconnected due to length limitation.


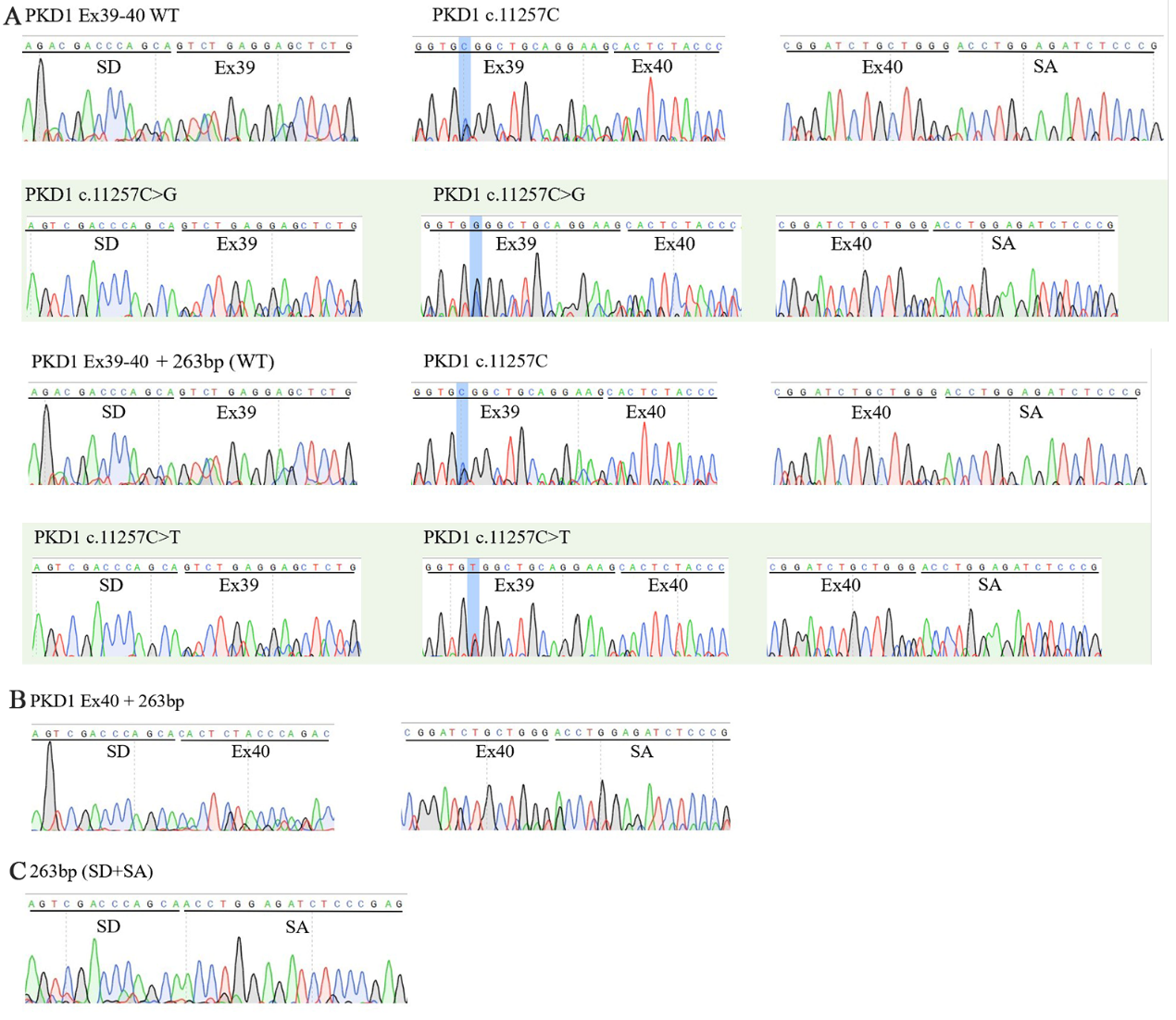


**Supplementary Figure S3. Sequencing diagrams of transcripts of variants on exon 39 of *PKD1.*** (A) The fragment contained the exon 39 and exon 40, flanked by two exons of the pSPL3 vector (SD and SA, 263bp). (B) The transcript comprised the exon 40 and pSPL3 vector sequence. (C) The segment included only the 263bp sequence of the pSPL3 vector. The blue boxes indicate the mutation sites. The sequencing results were continuous, but the presentation was disconnected due to length limitation.


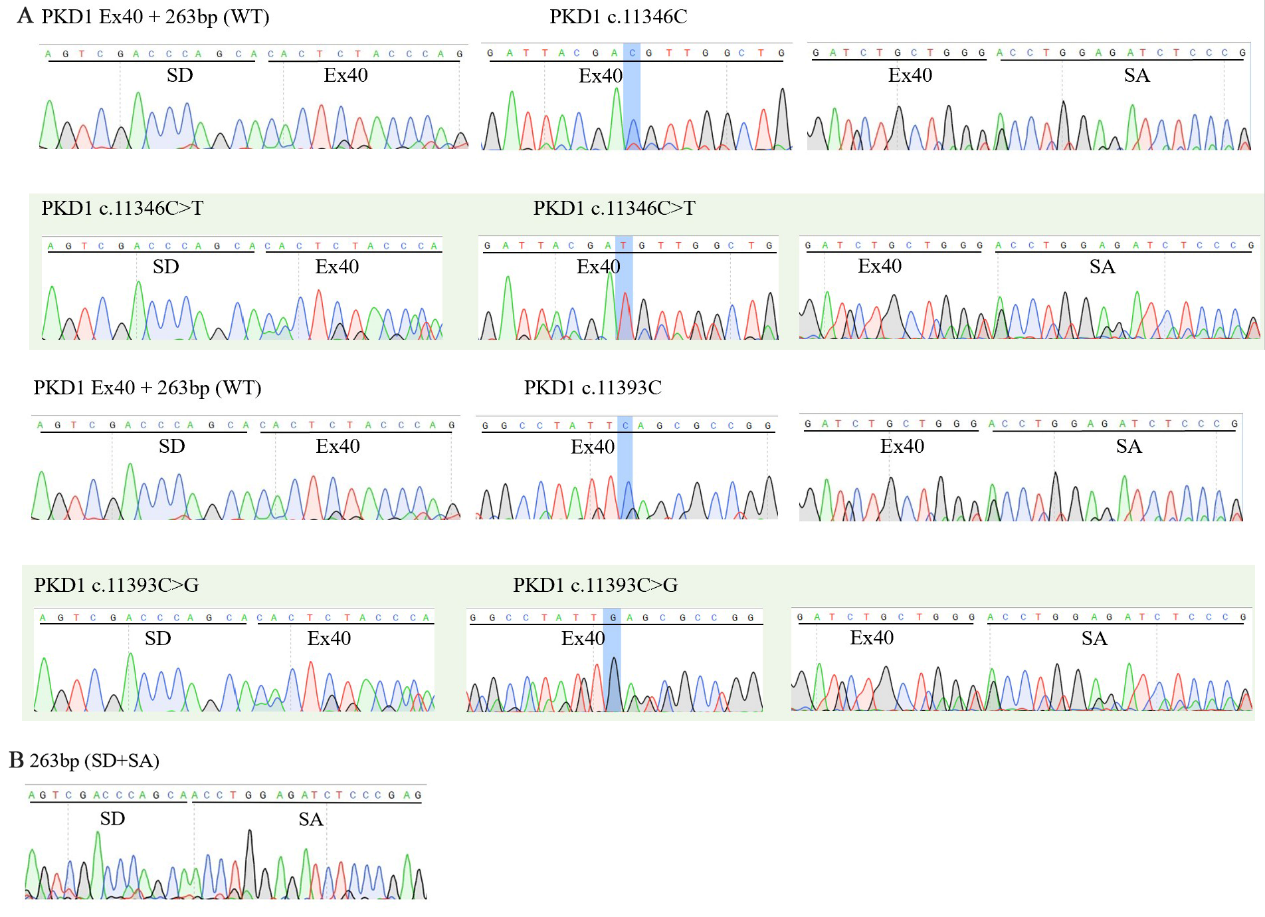


**Supplementary Figure S4. Sequencing diagrams of transcripts of variants on exon 40 of *PKD1.*** (A) The fragment contained the exon 40 flanked by two exons of the pSPL3 vector (SD and SA, 263bp). (B) The segment included only the pSPL3 vector sequence. The blue boxes indicate the mutation sites. The sequencing results were continuous, but the presentation was disconnected due to length limitation.


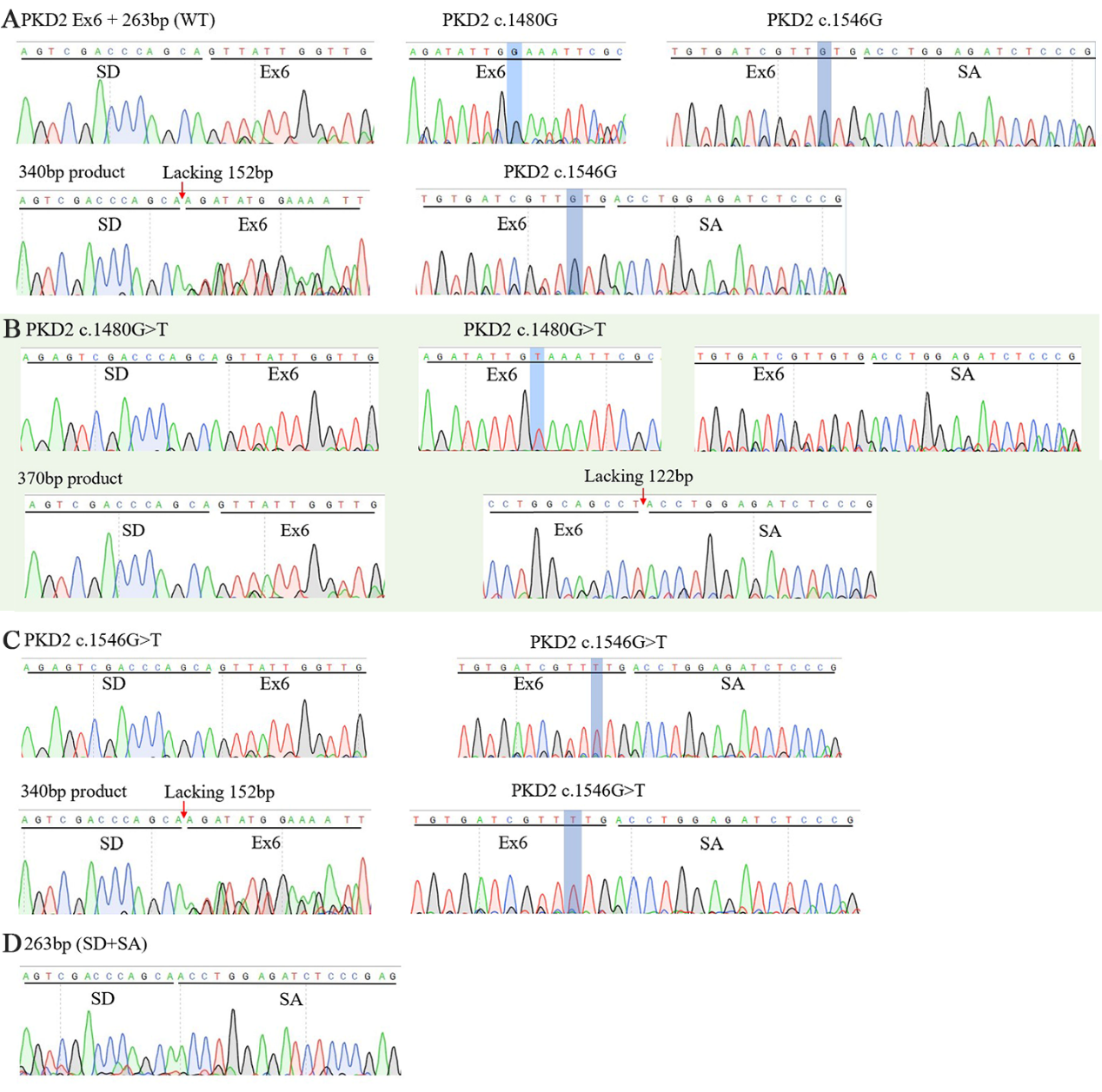


**Supplementary Figure S5. Sequencing diagrams of transcripts of variants on exon 6 of *PKD2.*** (A) Two transcripts of WT minigene of *PKD2* Ex6: the larger fragment contained the exon 6 flanked by two exons of the pSPL3 vector (SD and SA, 263bp), and smaller one (340bp product) consisted of an incomplete exon 6 lacking 152bp from the 5′ end. (B) Two fragments of the minigene of variant c.1480G>T: the larger fragment contained the exon 6 with variant c.1480G>T, and smaller one (370bp product) comprised a 122bp segment of exon 6 deletion from the 3' end. (C) Two transcripts of the minigene of variant c.1546G>T: the larger fragment contained the exon 6 with variant c.1546G>T, and smaller one (340bp product) consisted of an incomplete exon 6 lacking 152bp from the 5′ end. (D) The segment included only the 3′ and 5′ pSPL3 exons. The blue boxes indicate the mutation sites. The sequencing results were continuous, but the presentation was disconnected due to length limitation.

**
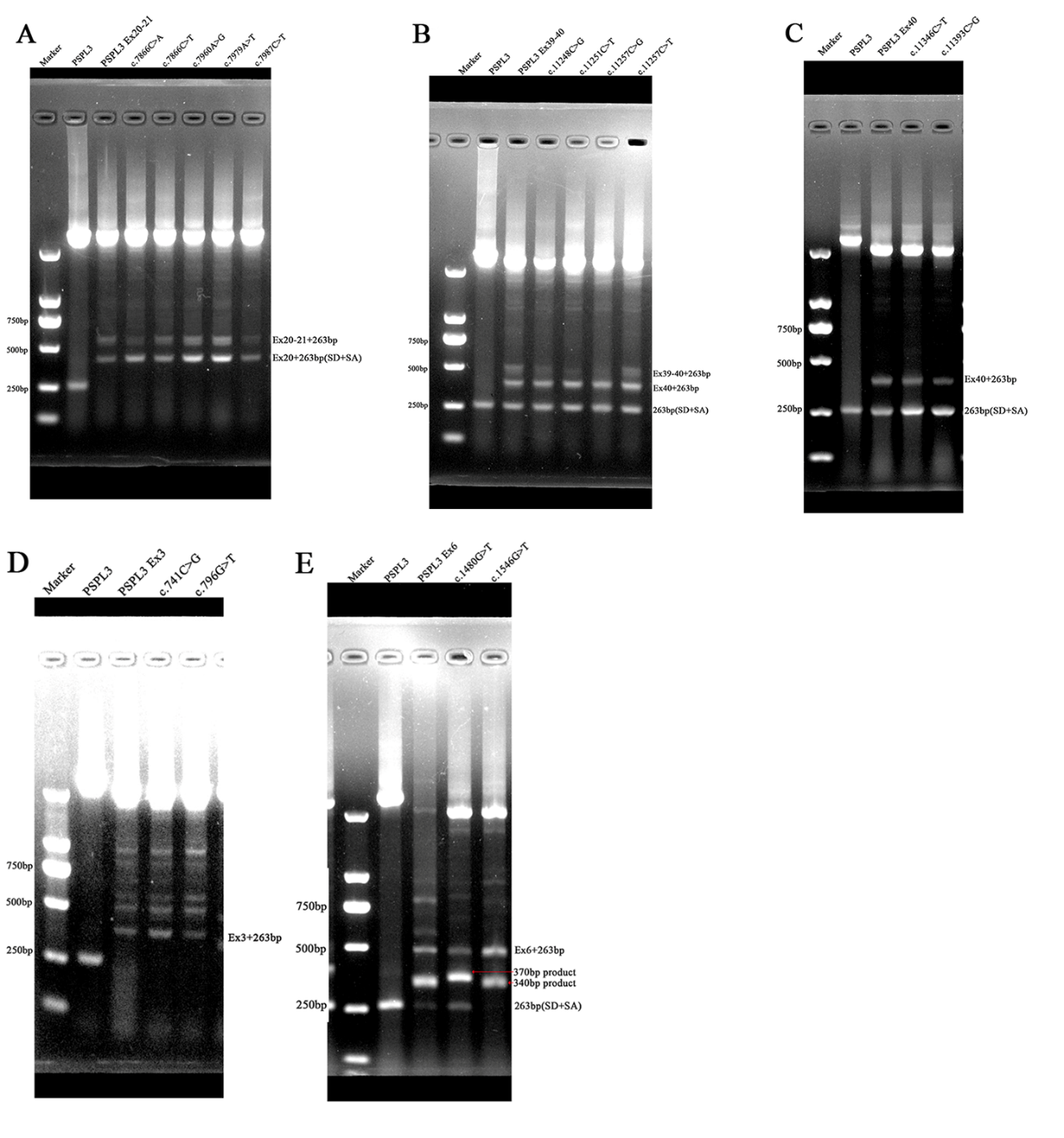
**

**Supplementary Figure S6. Complete AGAR gel figure.** (A) The AGAR gel figure of the variants in exon 21 of *PKD1.* (B) The AGAR gel figure of the variants in exon 39 of *PKD1.* (C) The AGAR gel figure of the variants in exon 40 of *PKD1.* (D) The AGAR gel figure of the variants in exon 3 of *PKD2.* (E) The AGAR gel figure of the variants in exon 6 of *PKD2.*
