# Supplementary material for: Identified eleven exon variants in PKD1 and PKD2 genes that altered RNA splicing by minigene assay
Source: BMC Genomics. 2023 Jul 19;24:407. doi: 10.1186/s12864-023-09444-9 (PMC10354997; doi:10.1186/s12864-023-09444-9)
Supplement: Supplementary file 8 — Supplementary Material 8 [file 12864_2023_9444_MOESM8_ESM.docx]

**Table S1** Primer sequences for amplifying exons.

| **Exons sequences (**5'-3'**)** |
| --- |
| *PKD1* EXON6-PSPL3-F CCG CTCGAG TGCCCACTCCCCTTCCTC  *PKD1* EXON6-PSPL3-R CTA GCTAGC TATGGCGTGCCCAGGAGT  *PKD1* EXON20-21-PSPL3-F CCG CTCGAG GAGACTGCCACCTGCTCACC  *PKD1* EXON20-21-PSPL3-R CTA GCTAGC GAACGGCTGAGGCTACTGAA  *PKD1* EXON37-38-PSPL3-F CCG CTCGAG GCCAGGGGTAGGCTACAG  *PKD1* EXON37-38-PSPL3-R CTA GCTAGC TGATGCCAGCAGCACCTA  *PKD1* EXON39-40-PSPL3-F CCG CTCGAG GCTGGCATCAGTAGGCAGAG  *PKD1* EXON39-40-PSPL3-R CTA GCTAGC AGAGGGGTGGCGTGGGTG  *PKD1* EXON40-PSPL3-F CCG CTCGAG GGCATCAGCCCTGCTCCCTA  *PKD1* EXON40-PSPL3-R CTA GCTAGC AGAGGGGTGGCGTGGGTG  *PKD2* EXON3-PSPL3-F CCG CTCGAG GGAAAGGAAGGCAAGGGTG  *PKD2* EXON3-PSPL3-R CTA GCTAGC GCACAGGCAAAGTTCTCA  *PKD2* EXON6-PSPL3-F CCG CTCGAG TTTTGCCGCTAGTTTGGG  *PKD2* EXON6-PSPL3-R CTA GCTAGC AATGCTGAGGAGATCAAAGACT |
