# Supplementary material for: Identified eleven exon variants in PKD1 and PKD2 genes that altered RNA splicing by minigene assay
Source: BMC Genomics. 2023 Jul 19;24:407. doi: 10.1186/s12864-023-09444-9 (PMC10354997; doi:10.1186/s12864-023-09444-9)
Supplement: Supplementary file 9 — Supplementary Material 9 [file 12864_2023_9444_MOESM9_ESM.docx]

**Table S2** Primer sequences for introducing mutations into exons.

| **Mutations sequences (**5'-3'**)** |
| --- |
| *PKD1* EXON6-1202-PSPL3-F TCTCCAGTGGTGCACCCGCTCT  *PKD1* EXON6-1202-PSPL3-R AGAGCGGGTGCACCACTGGAGA  *PKD1* EXON6-1248-PSPL3-F TTCCCTGGCAATGGGCACTGCTA  *PKD1* EXON6-1248-PSPL3-R TAGCAGTGCCCATTGCCAGGGAA  *PKD1* EXON20-21-7866CA-PSPL3-F CCAGTAAGAGCGGGCCCTGGA  *PKD1* EXON20-21-7866CA-PSPL3-R TCCAGGGCCCGCTCTTACTGG  *PKD1* EXON20-21-7866CT-PSPL3-F CCAGTATGAGCGGGCCCTGGA  *PKD1* EXON20-21-7866CT-PSPL3-R TCCAGGGCCCGCTCATACTGG  *PKD1* EXON20-21-7960-PSPL3-F TGTCCCTGGGGGTCCACACTGT  *PKD1* EXON20-21-7960-PSPL3-R ACAGTGTGGACCCCCAGGGACA  *PKD1* EXON20-21-7979-PSPL3-F ACTGTGGATGTCATCCAGCA  *PKD1* EXON20-21-7979-PSPL3-R TGCTGGATGACATCCACAGT  *PKD1* EXON20-21-7987-PSPL3-F ATGACATCCAGTAGATCGCTG  *PKD1* EXON20-21-7987-PSPL3-R CAGCGATCTACTGGATGTCAT  *PKD1* EXON37-38-11025-PSPL3-F AGAGCCTCCTAGTGTACATG  *PKD1* EXON37-38-11025-PSPL3-R CATGTACACTAGGAGGCTCT  *PKD1* EXON37-38-11119-PSPL3-F GCCATCAAGTAGGAGCTGCA  *PKD1* EXON37-38-11119-PSPL3-R TGCAGCTCCTACTTGATGGC  *PKD1* EXON39-40-11248-PSPL3-F ACGGCTGGGGCAGGTGCGGCT  *PKD1* EXON39-40-11248-PSPL3-R AGCCGCACCTGCCCCAGCCGT  *PKD1* EXON39-40-11251-PSPL3-F TGCGGTAGGTGCGGCTGCA  *PKD1* EXON39-40-11251-PSPL3-R TGCAGCCGCACCTACCGCA  *PKD1* EXON39-40-11257CG-PSPL3-F AGGTGGGGCTGCAGGAAGGT  *PKD1* EXON39-40-11257CG-PSPL3-R ACCTTCCTGCAGCCCCACCT  *PKD1* EXON39-40-11257CT-PSPL3-F AGGTGTGGCTGCAGGAAGGT  *PKD1* EXON39-40-11257CT-PSPL3-R ACCTTCCTGCAGCCACACCT  *PKD1* EXON40-11346-PSPL3-F AGCGATTACGATGTTGGCTG  *PKD1* EXON40-11346-PSPL3-R CAGCCAACATCGTAATCGCT  *PKD1* EXON40-11393-PSPL3-F CCTATTGAGCGCCGGATCT  *PKD1* EXON40-11393-PSPL3-R AGATCCGGCGCTCAATAGG  *PKD2* EXON3-741-PSPL3-F CCAATGTGTAGTACTACACCC  *PKD2* EXON3-741-PSPL3-R GGGTGTAGTACTACACATTGG  *PKD2* EXON3-796-PSPL3-F GTCCAAAACGTAGAAAAC  *PKD2* EXON3-796-PSPL3-R GTTTTCTACGTTTTGGAC  *PKD2* EXON6-1480-PSPL3-F GGAAGAGATATTGTAAATTCGC  *PKD2* EXON6-1480-PSPL3-R GCGAATTTACAATATCTCTTCC  *PKD2* EXON6-1546-PSPL3-F GTGATCGTTTTGGTAGGTTTG  *PKD2* EXON6-1546-PSPL3-R CAAACCTACCAAAACGATCAC |
